# Erythrocyte membrane with CLIPPKF as biomimetic nanodecoy traps merozoites and attaches to infected red blood cells to prevent *Plasmodium* infection

**DOI:** 10.1186/s12951-022-01709-x

**Published:** 2023-01-16

**Authors:** Zhouqing He, Chuyi Yu, Ziyi Pan, Xiaobo Li, Xiangxiang Zhang, Qijing Huang, Xingcheng Liao, Jiaoting Hu, Feng Zeng, Li Ru, Wanlin Yu, Qin Xu, Jianping Song, Jianming Liang

**Affiliations:** 1 Artemisinin Research Center, The First Affiliated Hospital, Guangzhou University of Chinese Medicine, Guangzhou, 510405 China; 2 Guangdong Provincial Hospital of Chinese Medicine, Guangzhou, 510120 China; 3 Key Laboratory of Smart Drug Delivery, School of Pharmacy, Ministry of Education, Fudan University, Shanghai, 201203 China

**Keywords:** Artemether, Erythrocyte membrane biomimetic nanomaterials, *pb*ANKA-infected malaria, Merozoites, Targeted delivery

## Abstract

**Background:**

Malaria remains a serious threat to global public health. With poor efficacies of vaccines and the emergence of drug resistance, novel strategies to control malaria are urgently needed.

**Results:**

We developed erythrocyte membrane-camouflaged nanoparticles loaded with artemether based on the growth characteristics of *Plasmodium*. The nanoparticles could capture the merozoites to inhibit them from repeatedly infecting normal erythrocytes, owing to the interactions between merozoites and heparin-like molecules on the erythrocyte membrane. Modification with a phosphatidylserine-targeting peptide (CLIPPKF) improved the drug accumulation in infected red blood cells (iRBCs) from the externalized phosphatidylserine induced by *Plasmodium* infection. In *Plasmodium berghei* ANKA strain (*pb*ANKA)-infected C57BL/6 mice, the nanoparticles significantly attenuated *Plasmodium*-induced inflammation, apoptosis, and anemia. We observed reduced weight variation and prolonged survival time in *pb*ANKA-challenged mice, and the nanoparticles showed good biocompatibility and negligible cytotoxicity.

**Conclusion:**

Erythrocyte membrane-camouflaged nanoparticles loaded with artemether were shown to provide safe and effective protection against *Plasmodium* infection.

**Graphical Abstract:**

**Figure:**
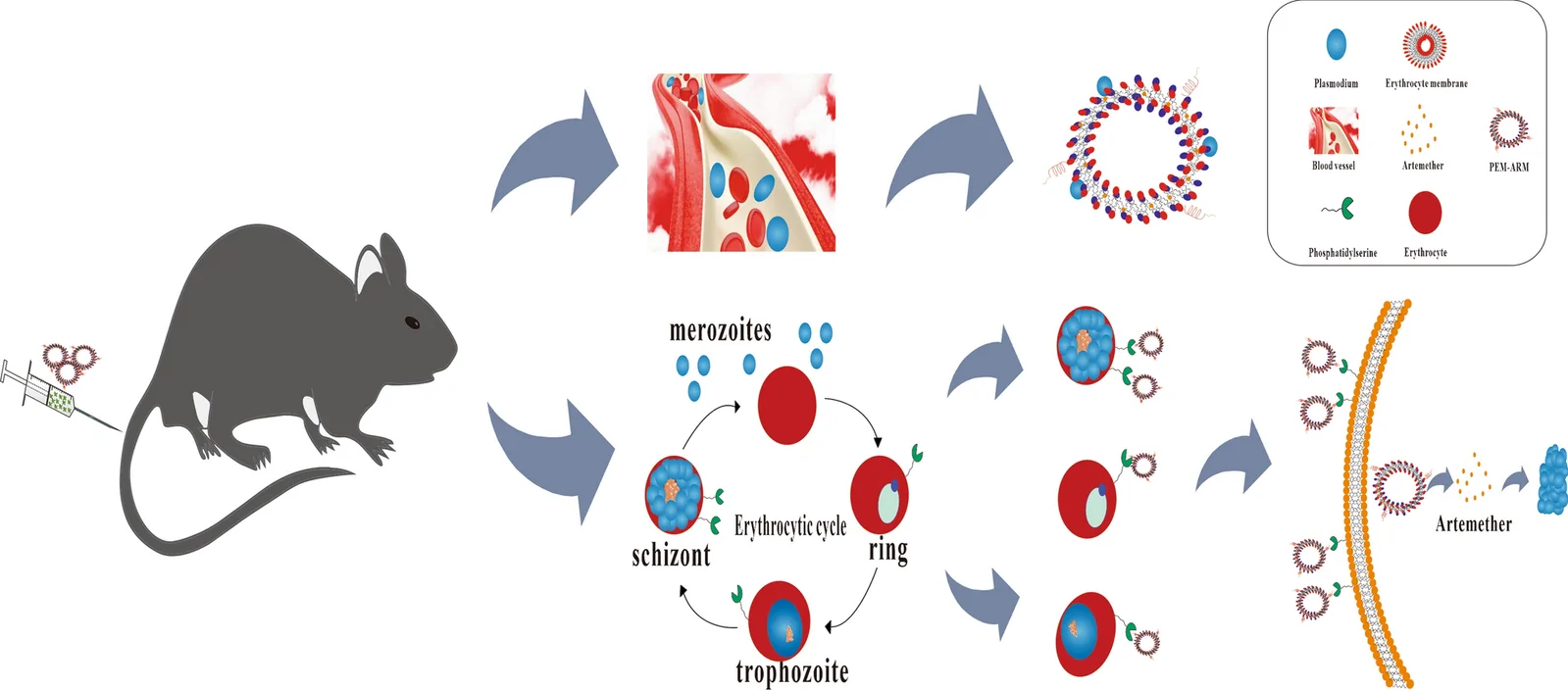

**Supplementary Information:**

The online version contains supplementary material available at https://doi.org/10.1186/s12951-022-01709-x.

## Introduction

Malaria is a vector-borne infectious disease that poses a serious health hazard to humans. Like tuberculosis and AIDS, it has been listed by the World Health Organization as an urgent global public health problem [1]. There are five species of protozoan *Plasmodium* parasites known to act as vectors of malaria in humans, including *P. vivax*, *P. malariae*, *P. falciparum*, *P. ovale*, and *P. knowlesi*. Among them, *P. falciparum* is the main cause of death and accounts for the majority of neonatal mortalities associated with low birth weight [2]. To date, the morbidity and mortality of malaria around the world have not been effectively controlled. In the long-term battle against malaria, artemisinin has historically been extracted from the Chinese medicinal herb *Artemisia annua*, and is a sesquiterpene lactone compound containing an internal peroxy bridge structure [3]. Compared with other available drugs such as chloroquine and hydroxyquine, artemisinin plays an essential role in the control of multiple stages of *Plasmodium* in the erythrocytic cycle [4, 5]. Despite the effectiveness of artemisinin, its clinical application was restricted by its low bioavailability, poor solubility, and short half-life [6]. Artemether (ARM) is one of the main derivatives of artemisinin with relatively stable chemical properties, and its anti-malarial effect is six times higher than that of artemisinin. However, its dosage is higher owing to its lack of targeting ability, conferring an increased risk of cardiotoxicity, embryotoxicity, and neurotoxicity [7]. Thus, ARM delivery through novel nano-biomimetic platforms remains a worthwhile challenge for overcoming the limitations of ARM.

During malaria infection, merozoites in the bloodstream invade normal erythrocytes and complete the process of development through the ring, trophozoite, and schizont stages [8]as a result of a series of host-pathogen interactions [9]. Thus, the key to capturing merozoites and preventing recurrent infection of normal erythrocytes is disturbing these interactions. One study showed that nanomimics of host cell membranes (prepared through a simple self-assembly procedure without post-formation modifications) could block invasion and expose invasive malaria parasites, resulting in a remarkable therapeutic effect [10]. In another study, immune engulfment was reduced following the conjugation of SIRPα on the surface of immune cells and an immunosuppressive red blood cell (RBC)-membrane protein CD47 [11].

**Fig. 1:**
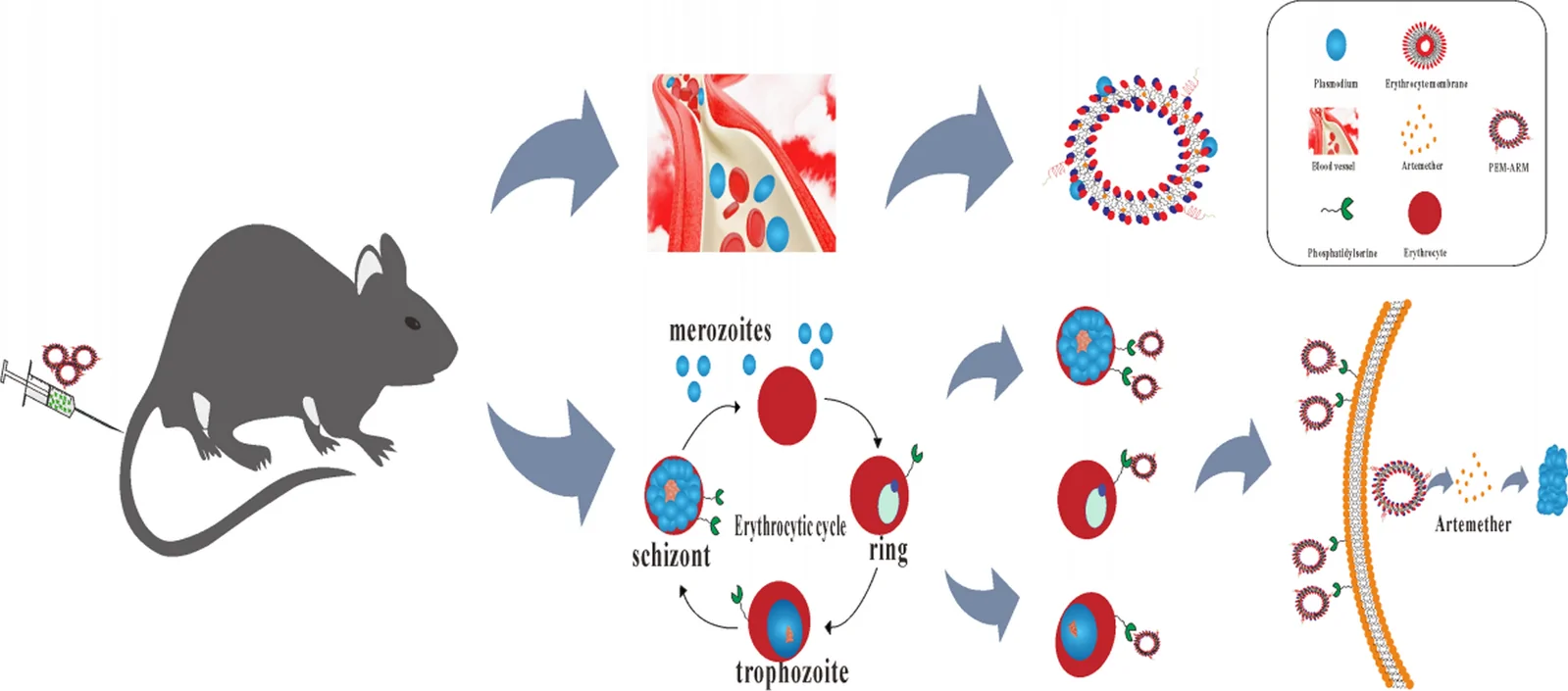
Schematic illustration of PEM-ARM. PEM-ARM was fabricated by surrounding the erythrocyte membrane with artemether liposomes modified with the CLIPPKF peptide. The two components of PEM-ARM made it capable of simultaneously targeting merozoites and infected erythrocytes in *pb*ANKA-infected mice

The membrane-bound lipid phosphatidylserine (PS), which is normally confined to the inner leaflet of the plasma membrane bilayer, redistributes and externalizes to the outer leaflet at the early stage of apoptosis in response to damage stimuli [12]. A study has shown that malaria-infected RBCs (iRBCs) can also lead to the exposure of PS, and the everted PS can be employed as a specific molecule to distinguish iRBCs from unaffected RBCs [13]. Based on the previously mentioned studies, we chose ARM as the therapeutic drug, prepared ARM liposomes (Lip-ARM), and utilized the long circulation of the natural erythrocyte membrane coating and its characteristic of not being scavenged by the immune system to produce erythrocyte membrane biomimetic ARM-loaded nanoliposomes (EM-ARM). Finally, the peptide CLIPPKF, which has a strong affinity to the everted PS on the surface of cells [14], was applied to modify EM-ARM and form PS-targeting peptide-modified biomimetic nanoliposomes (PEM-ARM). We aimed to endow PEM-ARM with dual targeting abilities for merozoites and iRBCs so it could be effective against multiple stages of *Plasmodium* (shown in Fig. 1). The dual targeting effects, therapeutic outcomes against malaria, and biological safety of PEM-ARM were evaluated in this study.

## Materials and methods

### Materials and reagents

Bovine serum albumin, phosphate buffer solution (PBS), trypsin, Roswell Park Memorial Institute (RPMI) 1640 Medium, Dulbecco’s Modified Eagle Medium (DMEM), DMEM/F-12, fetal bovine serum and penicillin & streptomycin & amphotericin B solution were purchased from Gibco, USA. CLIPPKF was purchased from Bank Peptide biological technology Co., Ltd, Hefei, China with the purity above 95%. Distearoyl phosphoethanolamine (DSPE)-PEG_2000_-Mal (Maleimide) was obtained from Laysan Bio Co., Arab, AL. Cholesterol (CHO), eggplant bottle, heparin sodium and Giemsa staining solutions were purchased from Titan Scientific Co., Ltd., Shanghai, China. Egg phosphatidylcholine (EPC), H_2_O_2_ and Tween-80 were purchased from J&K Chemical Ltd., Shanghai, China. Nile red (NR) and DAPI dye were from Beijing Fanbo Science and Technology Co., Ltd., Beijing, China. Percoll separation solution was obtained from Yibaiju Economic and Trade Co., Ltd., Shanghai, China. Annexin V-Cy5 and 10 × Annexin V binding buffer were from BioVision Inc, USA. Artemether, insulin and SYBR Green I was provided by Dalian Meilun Biology Technology Co., Ltd., Dalian, China. Carboxyfluorescein diacetate succinimidyl ester (CFDA-SE) was purchased from Beyotime Institute of Biotechnology, Beijing, China. The Hoechst 33,342 (HO), thiazole orange (TO), 5-(dimethylthiazol-2-yl)-2,5-diphenyltetrazolium bromide (MTT), hematoxylin-eosin (H&E) staining kit, ethylenediaminetetraacetic acid (EDTA), bicinchoninic acid (BCA) assay kit and DAB chromogenic kit were purchased from Sigma Aldrich, USA. Zoletil™ 50 and paraformaldehyde were purchased from Youchong Biological Technology Co., Ltd., Guangzhou, China. Anti-mouse TNF and IL-6, Streptavidin HRP were purchased from Shanghai Baiye Biotechnology Center, Shanghai, China. CellROX™ Deep Red Reagent, MitoTracker™ Deep Red FM, Texas Red®-X-conjugated WGA and TUNEL cell apoptosis detection kit were from Thermo Fisher Scientific, USA. Tissue autofluorescence quencher (AutoFluo Quencher) was purchased from Beijing Pulilai Gene Technology Co., Ltd., Beijing, China. Sodium citrate, xylene, physiological saline and hydrochloric acid ethanol were purchased from Sinopharm Chemical Reagent Co., Ltd., China. Dimethyl sulfoxide (DMSO), dichloromethane, tetrahydrofuran, anhydrous ethanol and triethylamine were purchased from Sigma-aldrich, Germany. Nuclepore hydrophilic membrane was purchased from GE Healthcare life science, Whatman, England. PVDF blotting membrane, antibody CD47, HRP-conjugated secondary antibody, and Omni-Ecltm ultrasensitive chemiluminescence detection Kit were gained from Shanghai Epizyme Biomedical Technology Co., Ltd.

### Cells and animals

The murine lung alveolar type II-like epithelial cell line (MLE-12) cells were purchased from the Institute of Biochemistry and Cell Biology, Chinese Academy of Sciences, Shanghai, China. and cultured under the condition recommended, i.e. DMEM/F-12 supplemented with 10% FBS, 0.2% insulin and 1% penicillin & streptomycin & amphotericin B solution in an incubator at 5% CO_2_ and 37 °C. Institute for Cancer Research (ICR) and C57BL/6 mice, female, 6 weeks of age, were purchased from Guangdong Medical Laboratory Animal Center, Guangzhou, China. The *pb*ANKA strain was kindly presented by Prof. Jian Li, Xiamen University, China. The animal study was approved by the Ethics Committee of Laboratory Animal Center, Guangzhou University of Chinese Medicine in accordance with the guidelines for the protection and welfare of animal subjects.

### Preparation of PEM-ARM

The Lip-ARM formulation was found to be 10 mg for 100 mg lipids and 10 mL PBS buffer, with an EPC-CHO molar ratio of 4:1. Briefly, The ARM, EPC and CHO were simultaneously dissolved in a 3 mL mixture solution of absolute ethanol and dichloromethane (ratio of volume was 5:1), then this solution was added dropwise slowly to PBS (45 °C, 10 mL, pH = 7.4) at a rate of 0.23 mL/min and continuously stirred on magnetic stirrer at 750 rpm. Finally the formulation was sonicated for 15 min (Scientz-IID, 200 Hz), subsequently extruded through a 200 nm nuclepore hydrophilic membrane using a LiposoFast extruder apparatus (Avestin, Ottawa, Canada) and residual organic solvents were removed by rotating evaporation to obtain Lip-ARM. The erythrocyte membrane (RBCm) was isolated as previously reported [15], and its protein was determined by BCA assay kit. The RBCm and Lip-ARM were ultrasonicated together and then passed through 200 nm Nuclepore hydrophilic membrane back and forth at least 20 times to prepare the EM-ARM with different protein concentrations (0.3, 0.6, and 0.9 mg mL^− 1^). Ultimately, the PEM-ARM was finished by a simple sonication and extrusion through a 200 nm Nuclepore hydrophilic membrane after putting appropriate amount of DSPE-PEG_2000_-CLIPPKF into the EM-ARM system.

### Characterization of PEM-ARM

Sodium dodecyl sulfate-polyacrylamide gel electrophoresis (SDS-PAGE) was ultilized to analyze the proteins of the PEM-ARM, RBCm and Lip-ARM, which were prepared in PBS and measured by using a BCA kit. The samples accompied with 5× loading buffer were heated at 100 °C for 10 min and 30 µg of each sample was added into a 10% gel. At the same time, 4 µL protein marker was joined as a control. Those samples were ran at 80 V for 30 min and 120 V for 2 h, stained with the Coomassie blue for 30 min, and washed 3 times before the observation.

The morphological characteristics of the Lip-ARM, EM-ARM and PEM-ARM were detected with the transmission electron microscope (TEM, Messtechnik, Germany). Pre-sonicated droplets containing the Lip-ARM, EM-ARM, or PEM-ARM were contacted with the copper grid for 60 s, negatively stained with uranyl acetate for 30 s and finally dried at room temperature. Moreover, the particle size, zeta potential and polydispersity index (PDI) were measured with the dynamic light scattering (Malvern, UK). Similarly, the stability of liposomes was evaluated in the PBS (pH = 7.4) by monitoring the size change for seven consecutive days, and stability in 10% fetal bovine serum was further explored by measuring the change of particle size and absorbance value at 600 nm wavelength within 24 h.

### Construction of *pb*ANKA-infected mouse malaria model

Taking *pb*ANKA as the model strain and ICR mouse as the passage mice. When the infection rate was 7–10%, as determined by the Giemsa staining described above, we utilized a disposable microvascular spotting tube to collect 20 µL blood from the tail tip of ICR mouse, and counted the total erythrocyte numbers under the microscope after diluting 100 times with physiological saline. The iRBCs number was the product of infection rate and the total erythrocyte numbers. Each C56BL/6 mouse accepted an intraperitoneal injection of 200 µL physiological saline containing 10^6^ iRBCs, and mock-infected mice accompanied with equal amounts of physiological saline as a control group.

### Capture of iRBCs

As previously mentioned [16], 4 µmol L^− 1^ HO and 100 ng mL^− 1^ TO were opted to offer an optimal staining of the DNA and RNA content of iRBCs. The HO and TO dyes were pre-dissolved in PBS and DMSO to prepare 4 mmol L^− 1^ and 1 mg mL^− 1^ storage solutions, respectively. These solutions were stored at -20 °C generally and diluted to recommended concentrations when we used. We collected the blood of *pb*ANKA-infected mice with different infection rates (5%, 10% and 15–20%) through the orbital venous plexus, centrifuged it at 700 ×*g* and washed 3 times, and then centrifuged it at 3200 ×*g* and washed 3 times again to purify the erythrocytes. NR-labeled liposomes and erythrocytes were co-incubated in 1640 medium containing 10% fetal bovine serum and predetermined concentrations of HO and TO for 75 min at 37 °C. After the incubation, erythrocytes were centrifuged again to remove residual dyes and medium and resuspended in 1 × PBS, of which components were classified by flow cytometry. We made quantitative analysis of the uptake in each group, and analyzed the differences statistically with the IBM SPSS Statistics 20.0.

To directly investigate the interaction of PEM-ARM with iRBCs, the confocal laser scanning microscopy (CLSM) was implemented. Briefly, the NR-labeled PEM-ARM and purifed erythrocytes incubated together in 1640 medium containing 10% fetal bovine serum and 4 µmol L^− 1^ HO for 75 min in a 37 ^o^C water bath. When the co-incubation finished, we carried out the centrifugal and washing operations and placed the erythrocytes in the binding buffer containing Annexin V-cy5 at 4 ^o^C for 30 min. Subsequently, 10 µL preheated glycerin gelatin was fell on the surface of a clean glass slide, and 30 µL suspended erythrocytes solution was added dropwise and mixed. We mounted the cover glass along the edge of the slide to avoid air bubbles during the process and observed the colocalization situation under the CLSM.

### Neutralization of merozoites

As previously reported with minor modification [17], 65% Percoll separation solution was employed to separate iRBCs from purified erythrocytes. The detailed procedure was as follows: The purified blood was diluted in 1 mL PBS, and added slowly to 5 mL 65% Percoll separation solution along the tube wall without stir. The mixture was centrifuged at 2500 ×*g* for 30 min at room temperature. Subsequently, the mature iRBCs were in the upper layer of the solution and re-collected into a new tube by using micropipette. We washed these mature iRBCs 3 times with PBS, put them in 2 mL deionized water, vortexed vigorously, and centrifuged at 800 ×*g* for 10 min to remove the red supernatant. The merozoites left in the bottom were continued to centrifuge, and washed twice until the supernatant was transparent. We resuspended the merozoites in PBS solution containing 0.1% glucose to maintain their viabilities.

NR-labeled liposomes were incubated with the merozoites in 1640 medium containing 10% fetal bovine serum and 4 µmol L^− 1^ HO, endocytosis levels of various liposome by merozoites were quantitatively construed by flow cytometry after 75 min incubation. It was worth mentioning that the uptake of NR-labeled EM-ARM with different RBCm protein concentrations (0.3, 0.6 and 0.9 mg mL^− 1^) by merozoites was comparatively tested, too. To intuitively figure out the affinity of PEM-ARM and merozoites, the NR-labeled PEM-ARM and merozoites were co-incubated in 1640 medium containing 10% fetal bovine serum and 4 µmol L^− 1^ HO at 37 °C for 75 min. We conducted the centrifugal and washing operations of merozoites in PBS solution containing 0.1% glucose, placed 10 µL liquid glycerin gelatin on the surface of a clean glass slide, and then added 30 µL resuspended merozoites solution dropwise. The coverslip was mounted along the edge of the slide to avoid air bubbles during the process, and the co-localization situation was examined under the CLSM.

Cytoadeherence assay of merozoites and normal erythrocytes was conducted to further explore the capacity of PEM-ARM to block merozoites adhesion, HO-labeled merozoites was employed to invade CFDA-SE-stained normal erythrocytes as bellow: The merozoites or red blood cells were incubated alone, with or without dye (HO or CFDA-SE, respectively), to prove the successful fluorescent dye-labeling; HO stained merozoites were then incubated with CFDA-SE stained normal erythrocytes or with erythrocytes and quantities of drugs (PBS, f-ARM, Lip-ARM, EM-ARM, or PEM-ARM) for 30 min, and the PBS group was served as a model group. We detected the fluorescence through flow cytometry and analyzed the ratio change of the Q_2_ area cells cluster statistically after the co-incubation.

### Therapeutic effect of PEM-ARM in vivo

To explore the anti-malaria effect of PEM-ARM in C57BL/6 mice, 50 mice (n = 10, female, 6 weeks, 15–20 g) accepted an intraperitoneal injection of 200 µL physiological saline containing 10^6^ iRBCs at day 0. The mice received an intravenous injection of free ARM preparation (f-ARM), Lip-ARM, EM-ARM, or PEM-ARM containing an equivalent ARM content of 2.5 mg kg^− 1^ at day 5 with an average infection rate was 5%, once every other day for seven consecutive days, mock-infected mice were used as control. We did not only record the survival rate, body weight change of mice, but also detected the infection rate by using flow cytometry at day 5, every other day until the termination of experiment. Mice were euthanized for the comparative study of blood routine and organ coefficients (organ weight to body weight, %) and their lung samples were harvested for terminal deoxynucleotidyl transferase dUTP nick end labeling (TUNEL) staining assay and immunohistochemical analysis.

### In vivo levels of ROS or mitochondrial activity evaluation

We put the enriched erythrocytes in 1640 medium containing 4 µmol L^− 1^ HO, 100 ng mL^− 1^ TO and 5 µmol L^− 1^ CellRox or 150 nmol L^− 1^ Mitotracker reagent after the seventh time of tail vein administration. Centrifugal and washing procedures were performed in PBS when the incubation was finished, we detected the levels of ROS or mitochondrial activity in the ring, trophozoite and schizont stages of iRBCs through flow cytometry as described at experimental section in the main text.

### Safety of PEM-ARM

MLE-12 cells were seeded in the 6 well plates with 5 × 10^4^ per well overnight, and treated with DMEM/F-12 containing HO and f-ARM, Lip-ARM, EM-ARM or PEM-ARM (50 µg mL^− 1^) labeled with NR for 75 min in an incubator at 5% CO_2_ and 37 °C. The cells were washed 3 times with PBS and observed under the fluorescence microscope. Subsequently, the cells were collected by trypsin digestion, washed 3 times by centrifugation at 1000 rpm at room temperature, resuspended in PBS, and quantitatively detected by flow cytometry. MTT assay was employed to evaluate the cytotoxicity of PEM-ARM to MLE-12 cells. Briefly, the cells were seeded in 96 well plates at a density of 1 × 10^4^ cells per well and cultured for 12 h. Then different concentrations drugs (100, 50, 25, 12.5, 6.25, 3.125, 1.5625, 0.78125, 0.390625, and 0 µg mL^− 1^) were added to the medium, and cells were incubated for another 48 h. At the endpoint of the incubation, MTT was added to test the cell viability. The principle organs (hearts, livers, spleens, lungs, brains, and kidneys) were fixed in 4% neutral paraformaldehyde, processed routinely into paraffin and sectioned at 5 μm. These sections were stained with hematoxylin and eosin, and inspected under a microscope.

### Statistical analysis

All data were expressed as mean ± standard error of the mean (SEM). Statistically significant differences were evaluated using Student’s t-tests or one-way analysis of variance (ANOVA). The Kaplan-Meier survival function was applied for survival analysis, and the Mauchly’s test of sphericity was used for analysis of variance of repeated measures data (IBM SPSS Statistics 20.0). *P*-values less than 0.05 were considered statistically significant.

## Results and discussion

### Preparation and characterization of PEM-ARM

**Fig. 2:**
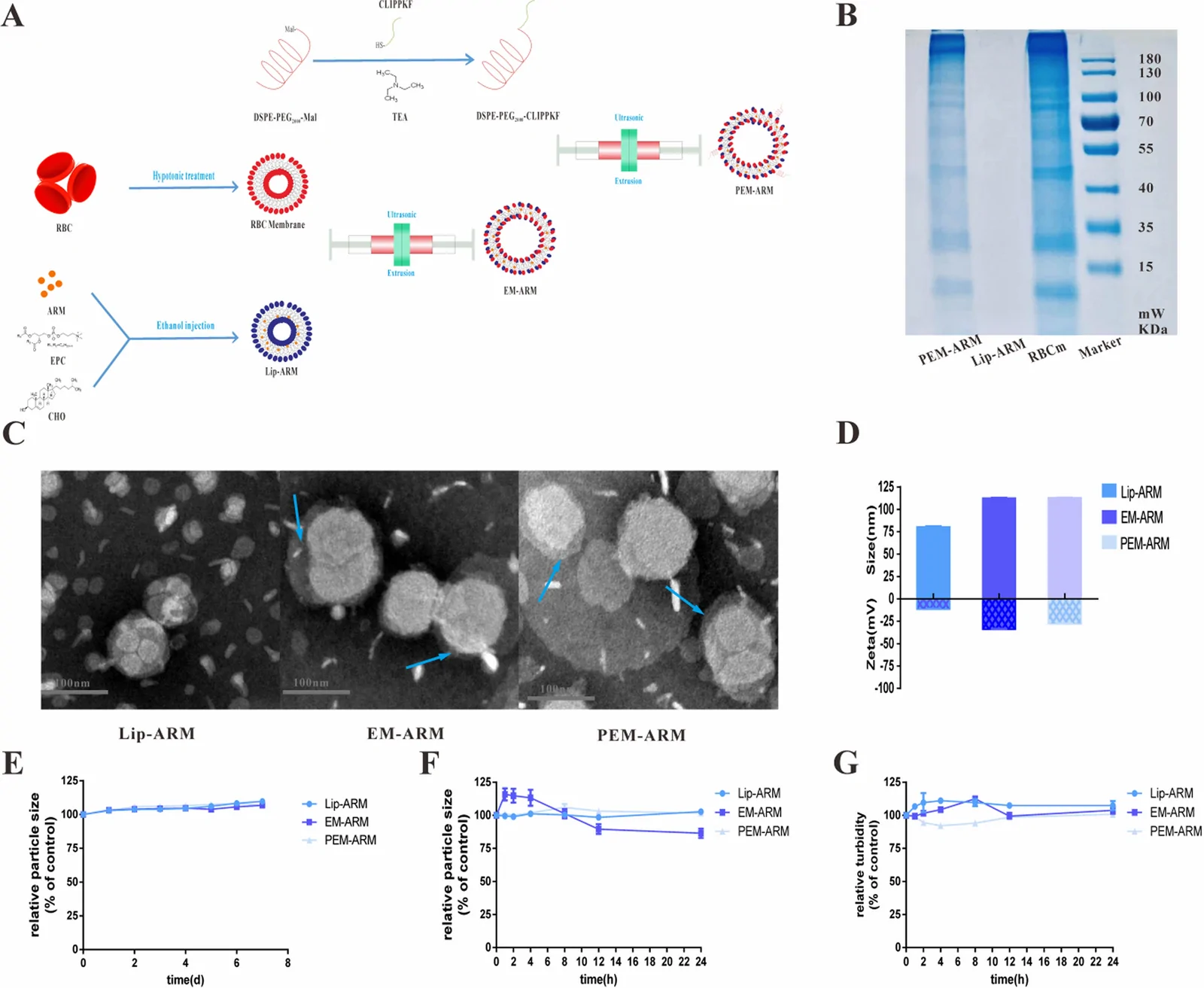
Preparation and characterization of PEM-ARM. **A** Schematic diagram of the preparation process of PEM-ARM. **B** Protein composition analyses of RBCm, Lip-ARM and PEM-ARM using SDS-PAGE. **C** Representative TEM images of Lip-ARM, EM-ARM, and PEM-ARM; erythrocyte membrane (blue arrow), scale bar: 100 nm. **D** Mean diameter and zeta potential of Lip-ARM, EM-ARM, and PEM-ARM. Data points represent mean ± s.d. (n = 3). **E** Stability of liposomes in water (n = 3). **F** Relative particle size and **G** relative turbidity in 10% serum of liposomes (n = 3)

To prepare PEM-ARM, RBC membranes were obtained as previously described [15], and the membrane protein concentration was quantitatively analyzed using the bicinchoninic acid method. The sulfhydryl-CLIPPKF complex was covalently bound to DSPE-PEG_2000_-Mal by the reaction of maleimide and sulfhydryl to form DSPE-PEG_2000_-CLIPPKF (Additional file 1: Fig. S1A) [18], which was confirmed by high-performance size exclusion chromatography (Additional file 1: Fig. S1B). The peak times of DSPE-PEG_2000_-Mal, CLIPPKF, and DSPE-PEG_2000_-CLIPPKF were 8.537 min, 10.784 min and 8.401 min, respectively. The peak time of the reactant was shortened from 8.537 min to 8.401 min as the molecular weight increased after the reaction, indicating the successful synthesis of DSPE-PEG_2000_-CLIPPKF. We prepared Lip-ARM using EPC, CHO, and ARM through the ethanol injection method. Then, RBCm was encapsulated on the surface of Lip-ARM using physical extrusion method to prepare EM-ARM. Finally, we modified EM-ARM with DSPE-PEG_2000_-CLIPPKF to construct PEM-ARM (Fig. 2A).

Next, SDS-PAGE was performed to help ensure RBC protein preservation on the PEM-ARM, which was crucial for the clearance of merozoites in the bloodstream. It showed that nearly all the major proteins on the RBC membrane were successfully translocated onto the PEM-ARM (Fig. 2B). Characterized protein CD47 was confirmed on the membrane surface using western blotting (Additional file 1: Fig. S2A). The asymmetric membrane distribution of glycoproteins, which reside exclusively on the extracellular side of erythrocytes under normal conditions, make them a good indicator of membrane orientation [19, 20]. In addition, the abundant negatively charged sialyl residues at the glycoprotein terminus endow a charge asymmetry across cellular membranes [21]. Fluorescently labeled wheat germ agglutinin (WGA) was recommended to characterize the glycoproteins, given that WGA is a protein that can selectively bind to N-acetylglucosamine and N-acetylsialic acid residues on the cell membrane [22]. It showed that Lip-ARM was hardly labeled by WGA, while EM-ARM or PEM-ARM could be labeled with WGA, and the fluorescence intensity was not significantly different from the corresponding RBCm (Additional file 1: Fig. S2B). The results indicated that the ultrasound extrusion process did not lead to visible glycoprotein loss or membrane turnover, which was critical for the proper functioning of the erythrocyte membrane. As shown in Fig. 2C, most of the liposomes were unilamellar, but a few were multichambered. The TEM image revealed Lip-ARM as a spherical vesicle structure with a diameter of approximately 80 nm. Both EM-ARM and PEM-ARM contained a lipid bilayer shell of approximately 9 nm in thickness, which was consistent with these reported previously [23]. Diameter and zeta potential, as measured by dynamic light scattering (Fig. 2D), revealed that the resulting PEM-ARM was approximately 30 nm larger than bare Lip-ARM with a small PDI, and a surface zeta potential interposed itself between Lip-ARM and EM-ARM. In vitro stability of nanoliposomes was determined by measuring the change in size over seven days. Stable nanoliposome size was observed throughout the study (Fig. 2E). Stability in the serum was further explored by measuring 24-hour changes in particle size and turbidity in 10% fetal bovine serum. The nanoparticles exhibited good stability with minor changes in size (Fig. 2F, G).

After confirming the structure of PEM-ARM, we investigated the EE and DL of ARM in the liposomes by the HPLC. It showed that the presence of CHO and EPC did not affect the normal absorption of ARM, suggesting that the method was highly specific, and the ARM content could be determined after liposome demulsification (Additional file 1: Fig. S3). We established the standard curve equation of ARM as: Y = 252.95 X + 0.0433, R^2^ = 0.9982. The peak area and concentration had a good linear relationship while the concentration of ARM was 0.025-0.5 mg mL^− 1^ (Additional file 1: Fig. S4). As calculated by the method established above, the mean EE and DL of ARM were approximately 93.4% and 4.0%, respectively (Additional file 1: Table S1).

### Capture of iRBCs

**Fig. 3:**
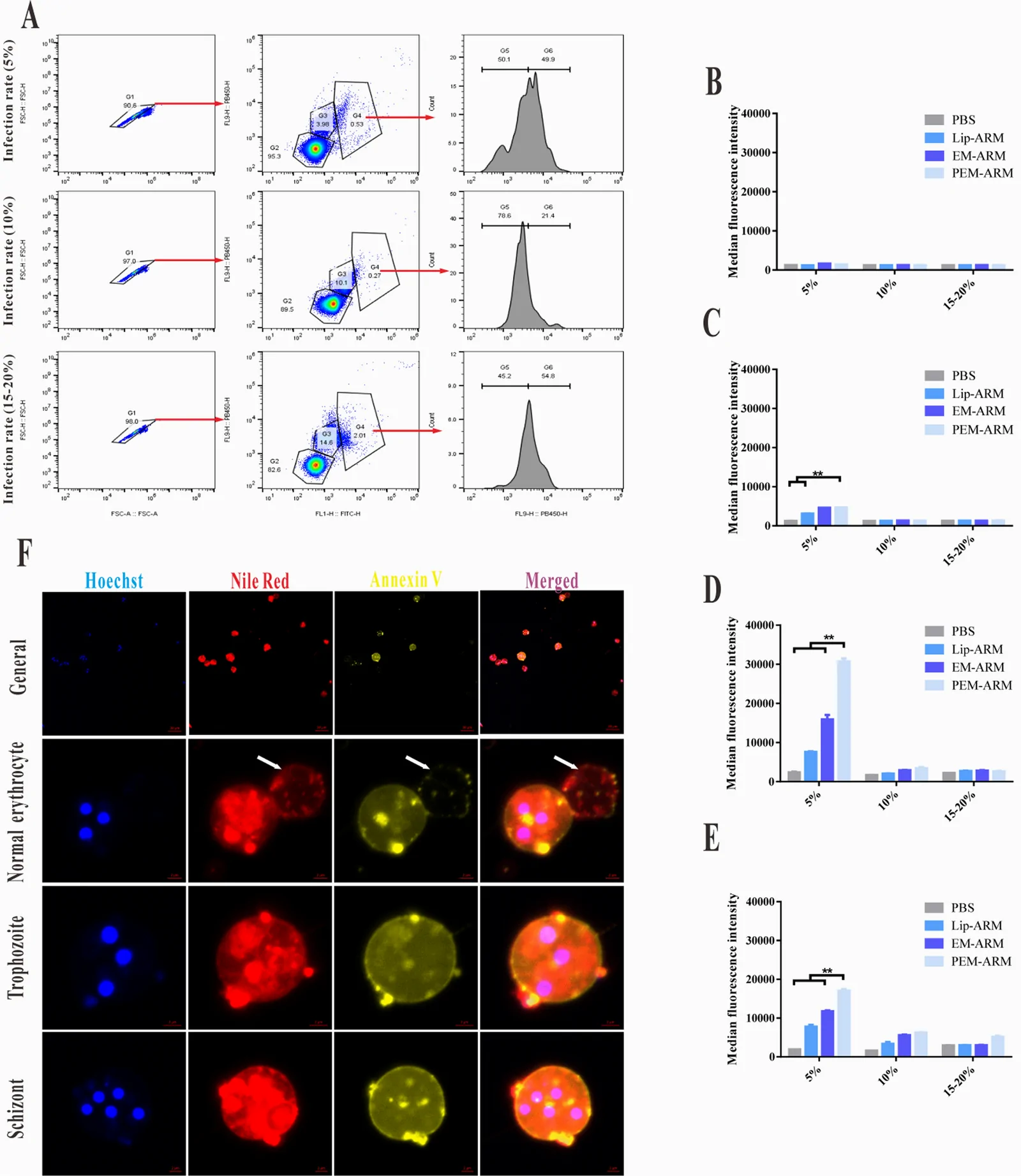
iRBCs-binding capacity in mice with malaria. **A** Analysis of the growth cycle of erythrocytes in *pb*ANKA-infected mice through the HO/TO double-staining method as assessed by flow cytometry. **B** Normal erythrocytes, **C** rings, **D** trophozoites, and **E** schizonts took up liposomes in mice with different infection rates (n = 3, ^*^ *P* < 0.05 and ^**^ *P* < 0.01). **F** Confocal fluorescent images of iRBCs (blue), PEM-ARM (red), and the everted PS on the surface of iRBCs (yellow) are shown. Normal erythrocyte (white arrow), scale bars: 20 μm in general; 2 μm in normal erythrocyte, trophozoite, and schizont

We used *pb*ANKA as the model strain and employed it to infect C57BL/6 mice, and the thin blood films of mice with different infection rates were treated with Giemsa staining to enable distinguishing iRBCs from normal erythrocytes using microscopy (Additional file 1: Fig. S5A and B). Taking blood from the orbital venous plexus, the differential centrifugation method was adopted to purify erythrocytes by removing nucleated components such as leukocytes and platelets. As previously reported [16], the HO/TO double-staining method was recommended to analyze intracellular DNA/RNA levels by flow cytometry, through which we classified the erythrocytes as follows: G2 (HO^−^/TO^−^) was the mass of a normal erythrocyte, G3 (HO^+^/TO^−^) represented the ring stage of iRBCs, and G4 (HO^+^/TO^+^) represented the trophozoite and schizont groups of iRBCs, which were further divided into G5 (< 4 N) for trophozoite and G6 (≥ 4 N) for schizont by DNA fluorescence intensity (Fig. 3A).

To comprehensively investigate the iRBCs-binding capacity of PEM-ARM in malaria infections, PEM-ARM labeled with NR dye was incubated with erythrocytes of different infection rates and treated with HO/TO dyes as mentioned above. Flow cytometry detection and quantification of G2 indicated little absorption of liposomes in normal erythrocytes (Fig. 3B). Analysis of G3 suggested that there was no statistically significant difference in the uptake of liposomes in mice with infection rates of 10% and 15–20%. In mice with an infection rate of 5%, in contrast to Lip-ARM, the median fluorescence intensity (MFI) of PEM-NR was significantly increased (Fig. 3C). Upon further observation and quantification of the absorptive capacity of G5 and G6 to PEM-ARM, we found that its MFI at 5% infection rate was significantly higher than that of other groups (Fig. 3D, E), and the MFI in PEM-ARM group was in the order of trophozoites > schizonts > rings > normal erythrocytes under the same conditions (Additional file 1: Fig. S6). Together, these results showed that PEM-ARM could effectively target iRBCs in the early stage of infection accompanied by notable drug internalization in the trophozoite stages.

Furthermore, upon labeling of the nuclear components of iRBCs (blue), PEM-ARM (red), and the everted PS on iRBCs (yellow) with HO, NR, and Annexin V-cy5 dyes, the CLSM imaging confirmed colocalization between iRBCs and PEM-ARM in general. It is noteworthy that compared with normal erythrocytes lacking a nucleus, trophozoites (< 4 N) and schizonts (≥ 4 N) contained more pronounced nuclei and had a larger quantity of everted PS, which tightly interacted with PEM-ARM. In addition, PEM-ARM could penetrate into iRBCs and co-localize with the nuclei of *Plasmodium*, which was rarely taken up by normal erythrocytes (Fig. 3F). As reported, malaria-infected RBCs can lead to the exposure of PS [13], and PEM-ARM could deliver the drug to iRBCs owing to the specific binding between CLIPPKF and the everted PS of iRBCs. These results demonstrated that PEM-ARM could efficiently work against iRBCs, as confirmed by flow cytometry.

### Neutralization of merozoites

**Fig. 4:**
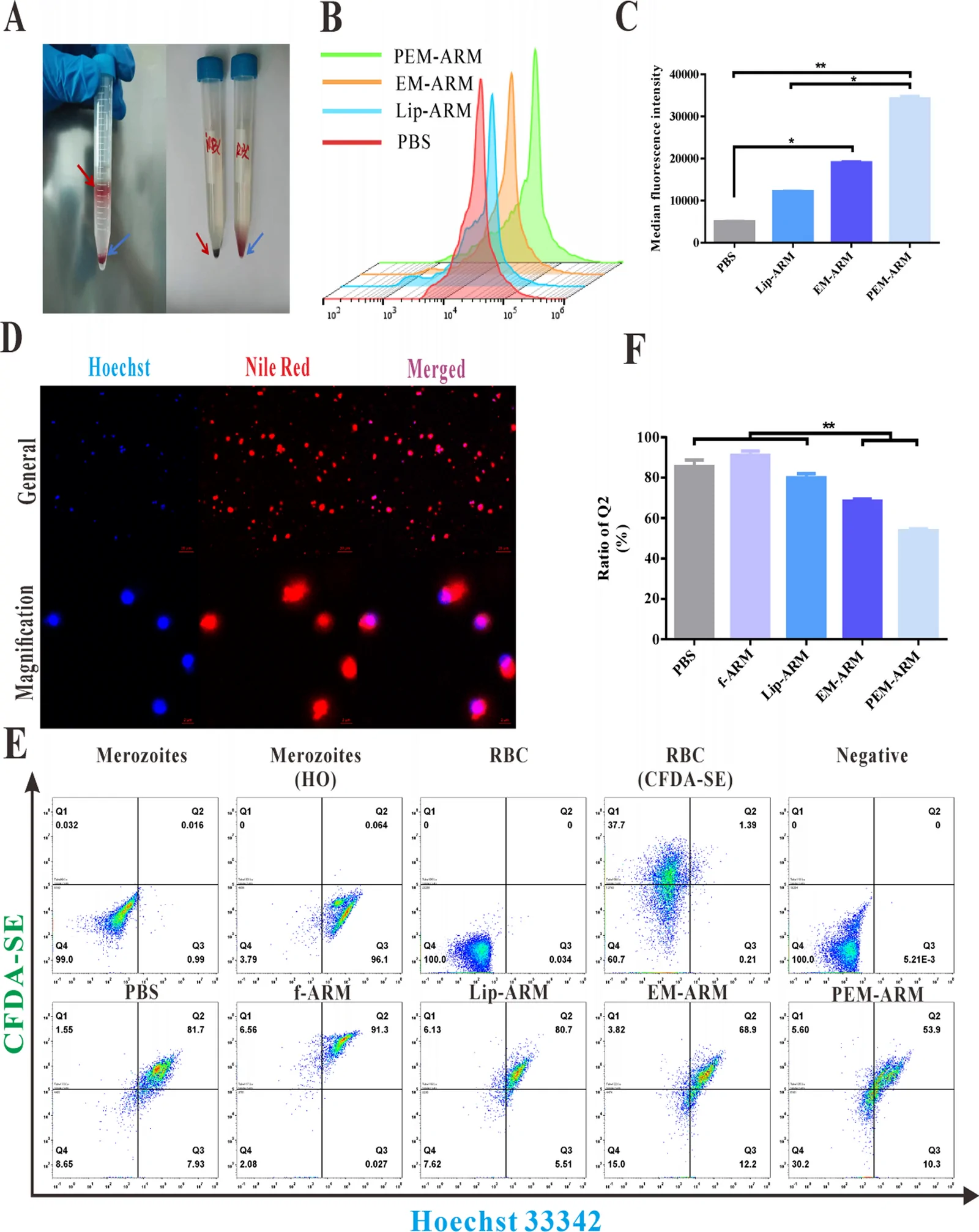
Neutralization of merozoites. **A** Purification of mature iRBCs with Percoll separation solution, illustrating iRBCs (red arrow) and normal erythrocytes (blue arrow). **B**, **C** Uptake of Lip-ARM, EM-ARM, and PEM-ARM by merozoites in *pb*ANKA-infected mice (n = 3, ^*^ *P* < 0.05 and ^**^ *P* < 0.01). **D** Confocal fluorescent images of merozoites (blue) and PEM-ARM (red); scale bar = 20 μm (general), scale bar = 2 μm (magnification). **E** Representative scatter plots of Hoechst 33,342/CFDA-SE analysis of the cytoadherence assay of merozoites and normal erythrocytes after drug treatment. **F** Percentage of Q_2_ area cells (n = 3, ^*^ *P* < 0.05 and ^**^ *P* < 0.01)

To effectively gather merozoites, as previously reported with minor modifications [17], 65% Percoll separation solution was utilized to purify mature iRBCs from the whole blood. As shown in Fig. 4A, unlike normal erythrocytes, iRBCs were in the upper layer of the solution following density gradient centrifugation. Further washing these cells with 1× PBS exhibited iRBCs that were brown-black and normal erythrocytes that were bright red.

As reported [24], merozoites have a strong affinity for the normal erythrocyte membrane. The PEM-ARM should present an affinity that is comparable to merozoites. To verify the hypothesis as mentioned above, the NR-labeled PEM-ARM was incubated with merozoites obtained from iRBCs through hypotonic hemolysis. Then, the fluorescence intensity of merozoites was measured and quantified. In contrast to non-membrane groups (PBS and Lip-ARM), the membrane-containing groups (EM-NR, PEM-NR) had significantly stronger fluorescence intensity, indicating that the normal erythrocyte membrane in liposomes was capable of capturing merozoites (Fig. 4B, C). Nevertheless, it was interesting to note that the uptake of EM-ARM with a membrane protein concentration of 0.6 mg mL^− 1^ was higher than that of both 0.3 mg mL^− 1^ and 0.9 mg mL^− 1^, indicating that the adsorption of merozoites by normal erythrocyte membrane could reach a plateau (Additional file 1: Fig. S7). Next, the interaction of NR-labeled PEM-ARM with HO-marked merozoites was observed under confocal laser scanning microscopy, and violet fluorescence was seen after colocalization. After magnification, we found that PEM-ARM had the ability to bind to merozoites (Fig. 4D).

To further examine whether PEM-ARM can inhibit merozoites from adhering to normal erythrocytes, CFDA-SE-stained normal erythrocytes were incubated with HO-stained merozoites or with merozoites and quantities of the drugs. The results showed that the negative group was a non-fluorescent zone, distributed in the Q_4_ area, while the cell clumps of the PBS group predominantly fell in the Q_2_ area, accounting for 85.37% on average. These results indicated that merozoites successfully invaded normal erythrocytes (Fig. 4E). Subsequently, statistical analysis of the ratio of Q_2_-area cells revealed that except for f-ARM, both EM-ARM and PEM-ARM decreased the proportion of merozoites invading RBCs to a certain extent, occupying 68.37% and 53.80%, respectively, with significant differences (Fig. 4F). Merozoite invasion, which is essential for parasite survival and proliferation, is a potential strategy for anti-malarial drugs. However, as reported [25], many first-line anti-malarial drugs, such as chloroquine and artemisinin, cannot inhibit merozoite invasion of the RBC, which is crucial for *Plasmodium* clearance. Merozoite invasion needs multiple receptor-ligand interactions between the erythrocyte and the merozoites [26], and in this study, most erythrocyte membrane proteins were retained in camouflaged nanoparticles. Thus, the camouflaged nanoparticles could bind merozoites like pore-forming toxins [27]. These results indicate that the PEM-ARM had a powerful merozoite-arresting effect that could block the merozoites from repeatedly infecting normal erythrocytes and potentially deterring the periodic attacks of malaria.

### Therapeutic effect of PEM-ARM in vivo

**Fig. 5:**
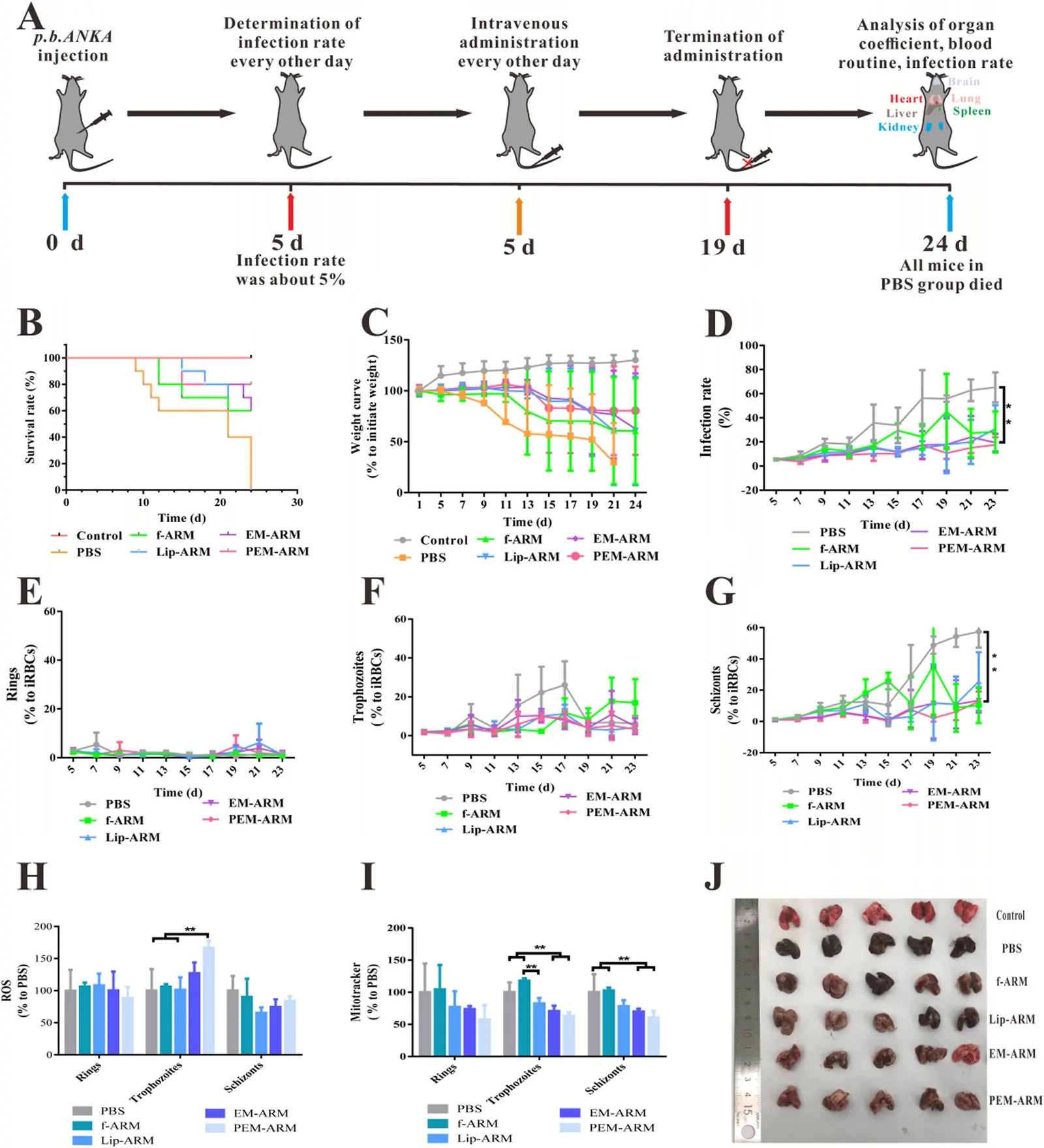
Therapeutic effect of PEM-ARM in vivo. **A** Sketch of the therapy regimen. **B** Survival curve of C57BL/6 mice exposed to *pb*ANKA infection. **C** Weight change curves of C57BL/6 mice. **D** Flow cytometry detection of infection rate in mice (n = 5, ^*^ *P* < 0.05 and ^**^ *P* < 0.01). **E** Ring, **F** trophozoite, and **G** schizont stages of iRBCs changed in proportion. (n = 5, ^*^ *P* < 0.05 and ^**^ *P* < 0.01). **H** Reactive oxygen species and **I** mitochondria activity analysis of iRBCs treated with different drugs (n = 3, ^*^ *P* < 0.05 and ^**^ *P* < 0.01). **J** Mice lungs infected with *pb*ANKA or treated with PBS, f-ARM, Lip-ARM, EM-ARM, or PEM-ARM, with mock-infected mice lungs as a control

In this study, C57BL/6 mice were utilized as a model to systemically evaluate the anti-malaria efficacy of PEM-ARM. As shown in Fig. 5A, mice weighing 15–20 g received an intraperitoneal injection of 200 µL physiological saline containing 10^6^ iRBCs at Day 0. The mice received an intravenous injection of f-ARM, Lip-ARM, EM-ARM, or PEM-ARM containing an equivalent ARM content of 2.5 mg·kg^− 1^ at Day 5 with an average infection rate of 5%, once every other day for seven consecutive days. Meanwhile, the infection rate of the mice was assessed by flow cytometry every other day until the termination of the experiment. Mock-infected mice were used as control. Kaplan-Meier survival analysis demonstrated that all mice in the *pb*ANKA-infected group (PBS-treated) reached humane clinical endpoints at Day 24 after the injection of *pb*ANKA (Fig. 5B). It was worth noting that compared with the PBS group, the median survival time of all groups administered with ARM was prolonged. Remarkably, in contrast to the f-ARM group, the PEM-ARM group also delayed the initial death time. In summary, PEM-ARM resulted in a significant reduction in mortality and an extended survival time. Change in body weight was another predictive indicator of malaria [28]. Mauchly’s test of sphericity found that the *pb*ANKA-challenged mice rapidly lost weight, whereas mock-infected mice gradually increased in body weight under the same ambient environment. Mice administered with PEM-ARM experienced a descending extent of weight loss after injection with *pb*ANKA (Fig. 5C), which was beneficial for their health. Additionally, the efficacy of inhibiting the growth of malaria parasites in the blood was assessed by comparing the growth rate of parasitemia [29]. The infection rate of *pb*ANKA-infected animals sharply rose after Day 11 with restraint, whereas the mice in the PEM-ARM and EM-ARM groups dampened parasitemia rate growth post-challenge for the mice injected with *pb*ANKA (Fig. 5D). Further subdivision of the various blood stages of infection with the malaria parasite revealed that PEM-ARM primarily attenuated the specific gravity of the trophozoite and schizont stages of iRBCs (Fig. 5E, F, G), which was propitious for the depression of pathogenic virulence and invasiveness. Trophozoite, the stage when hemoglobin catabolism is maximal [30], releases toxic-free heme and results in a state of oxidative threat, where the process remains until the infected red cell bursts after the schizont stage. Thus, trophozoites of blood-stage malaria have been reported as a potential target for the treatment and prevention of malaria [31]. In this study, PEM-ARM significantly reduced the proportion of trophozoites and merozoites, as well as inhibited rupture and subsequent replication and proliferation. This PEM-ARM targeting mechanism, consistent with iRBC targeting, enhanced its anti-malarial efficacy.

Oxidative stress has been considered a self-protective mechanism after malaria infection. On one hand, the immune system activated by the parasite releases free radicals such as reactive oxygen species (ROS) [32]; on the other hand, the free radicals generated from ARM can combine to the malaria parasite’s protein, inactivating the protein and inducing the death of the malaria parasite [33]. Hence, the levels of ROS in iRBCs can be served as a fundamental index for estimating the therapeutic efficacy of malaria treatments. It should be considered that PEM-ARM chiefly diminished the proliferation of *Plasmodium* by stimulating an augmentation of ROS in the trophozoite stages (Fig. 5H). *Plasmodium* blood stages have a primarily glycolytic energy metabolism. Yet, they retain mitochondrial electron transport chain complexes II through IV, and their mitochondria remain indispensable in energy metabolism [34]. Thus, mitochondrial activity can be regarded as another useful index. In the trophozoite and schizont stages of iRBCs, relative to the PBS and f-ARM groups, EM-ARM and PEM-ARM manifested a strong downregulatory effect on mitochondrial activity (Fig. 5I).

For endpoint analysis, mice lungs were obtained for TUNEL staining and immunohistochemical analysis. Lungs from mock-infected mice were flesh red, elastic, and non-grainy, with a smooth surface and a normal size and shape, while those of *pb*ANKA-challenged mice were dull, had a rough and matte surface, showed signs of stress, and had a larger size and abnormal shape (Fig. 5J). Pulmonary pathologic changes observed by TUNEL staining revealed that *pb*ANKA caused apparent cell apoptosis in the lungs, whereas no evident cell apoptosis was observed following sufficient EM-ARM or PEM-ARM treatment (Additional file 1: Fig. S8A and B). Malaria-associated acute respiratory distress syndrome (MA-ARDS), as a fatal complication [35], causes adamant inflammation, inflammatory cell infiltration, and hemozoin deposition [36]. Immunohistochemical analysis results further supported the notion that compared with *pb*ANKA-infected mice, PEM-ARM successfully inhibited the production of TNF-α and IL-6 and the deposition of hemozoin (Additional file 1: Fig. S9). As suggested [37], the iRBCs not only escape the clearance of macrophages, but they also harvest additional benefits of promoting their growth after adherence to normal lung tissues, which was a positive determinant for the occurrence of MA-ARDS [38]. Normal lung tissues were stained with HO33342 co-incubated with Green I-marked iRBCs treated with different drugs, and the binding levels were quantified using a fluorescence microscope. As shown in Additional file 1: Fig. S10A and B, a large amount of iRBCs were sequestered in lung tissue, while the amount of iRBCs attached to normal lung tissues greatly decreased after PEM-ARM administration. The *P. falciparum*-infected erythrocytes are known to adhere to lung tissue in a CD36-dependent manner [39, 40], and PS-expressing cell membrane is an important ligand of CD36 [41]. In this study, PEM-ARM targeted iRBCs by interacting with the PS on the cell membrane (Fig. 3). We found that PEM-ARM successfully blocked externalized PS and prevented CD36-mediated sequestration of iRBCs in lung tissue. The results above indicated that PEM-ARM inhibited iRBC adhesion in lung tissue, down-regulated TNF-α and IL-6 levels, and further reduced iRBC-induced lung damage.

Malaria-related pernicious anemia contributes to another crucial cause of death [42]. It is essential to make some deductions about its significance in parasite infection after administration. Whole blood cell analysis in mice showed that all groups infected with *Plasmodium* suffered from different degrees of anemia compared with mock-infected mice; this was shown by the numbers of RBCs, hemoglobin, and hematocrit. Except for the EM-ARM and PEM-ARM administration groups, there were significant differences between mock-infected mice and non-membrane groups (f-ARM and Lip-ARM). Additionally, platelet counts in mice that were *pb*ANKA*-*infected or *pb*ANKA*-*infected with drugs administration decreased, This may be relative to malarial thrombocytopenia, which is hypothesized to be driven by platelet activation (Additional file 1: Fig. S11) [43]. The organ coefficients of the vital organs in mice, such as the liver, spleen, lungs, brain, kidneys and heart, were further studied, showing that the liver and spleen were enlarged. Compared with *pb*ANKA*-*infected mice, grades of liver enlargement in mice treated with PEM-ARM treatment were attenuated (Additional file 1: Fig. S12A and B), indicating that liver swelling could be reversible in a short time through the timely elimination of infection [44]. The lungs showed evidence of irregular pulmonary edema, contributing to an increase in the weight of the lungs (Additional file 1: Fig. S12C). The hearts and kidneys were slightly swollen, and mice with PEM-ARM therapy after challenge with *pb*ANKA exhibited ameliorated swelling in comparison with the PBS groups, which may be attributed to the advancement of hemodynamics (Additional file 1: Fig. S12D and E). The brain-to-body ratio of mice in the PBS group was higher than that of other groups, suggesting the presence of cerebral malaria (Additional file 1: Fig. S12F). Together, these in vivo results present promising therapeutic effects of PEM-ARM against *pb*ANKA infections and suggest the potential clinical applications.

### Safety of PEM-ARM

**Fig. 6:**
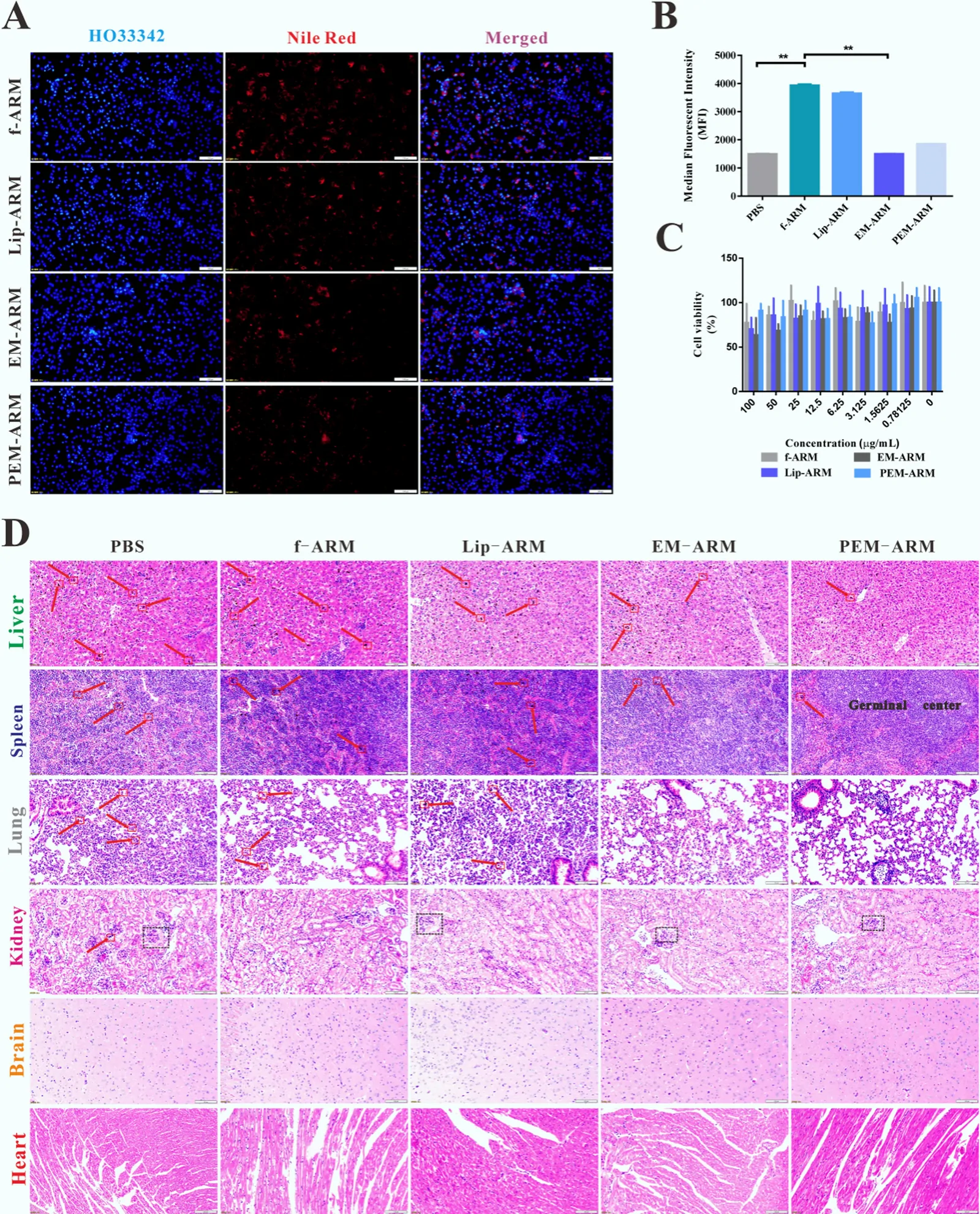
Characterizations of several measures of the safety of PEM-ARM. **A** Fluorescence images and **B** Median fluorescence intensity charts of MLE-12 cellular uptake after liposome treatment. Scale bar = 200 μm. Cell nuclei and the liposomes were labeled with HO (blue) and NR (red), respectively (n = 3, ^*^ *P* < 0.05 and ^**^ *P* < 0.01). **C** MTT assay after MLE-12 cells were incubated with various concentrations of liposomes (n = 6, ^*^ *P* < 0.05 and ^**^ *P* < 0.01). **D** H&E-stained slices of the liver, spleen, lung, kidney, brain, and heart. Scale bar = 200 μm, hemozoin (red arrow), glomerular capillary congestion sign (black frame)

Safety and biocompatibility are major considerations for nanomaterials used in biomedical applications [45]. In this study, mouse lung epithelial type II cells (MLE-12 cells) were used as normal cells to assess the toxicity of PEM-ARM, and we found that PEM-ARM was taken up less by MLE-12 cells (Fig. 6A, B) and did not noticeably decrease cell viability (Fig. 6C). Because of the outstanding mechanical stability of the RBC membrane, the hemolysis test has become a favorable indicator for screening out the cytotoxicity of various compounds [46]. Utilizing water as a positive control group, the hemolytic rate of PEM-ARM was less than 5%, which was similar to the negative group (PBS), suggesting good blood compatibility (Additional file 1: Fig. S13). To investigate the in vivo distribution after administration of NR-labeled liposomes in *pb*ANKA-challenged mice, we gathered the isolated organs (heart, liver, spleen, lungs, and kidneys) when mice were sacrificed at 24 h. The nanoparticles distribution in tissues was observed through the near-infrared fluorescence in vivo imaging system, it showed that PEM-ARM did not massively accumulate in the liver or spleen, indicating extremely low potential toxic side effects (Additional file 1: Fig. S14). Furthermore, we tested relevant serum biochemical parameters to ensure that no toxic effects existed 24 h after administration. As shown in Additional file 1: Fig. S15, there were no significant differences between the PBS group and administration groups in the contents of aspartate aminotransferase (AST), alkaline phosphatase (ALP), creatinine (CRE), albumin (ALB), total protein (TP), and the ratio of ALB to TP. Besides, the alanine aminotransferases (ALT) and urea were remarkably decreased in biomimetic membrane nanoparticles (EM-ARM and PEM-ARM groups), suggesting that they may have a role in protecting liver and kidney functions. Results after H&E staining revealed that large amounts of hemozoin were visible in the liver, spleen, and lungs. Hepatocellular vacuole-like denaturation, critical damage to the splenic germinal center, alveolar collapse with severe pulmonary consolidation, and eosinophil infiltration in the lumen indicated parasite infection. After intravenous administration, the drugs (f-ARM, Lip-ARM, EM-ARM, and PEM-ARM) sequentially reduced the deposition of malaria pigments in the lungs, liver, and spleen. Most of the alveoli maintained normal luminal morphology, and the pulmonary consolidation area was substantially diminished. Notably, PEM-ARM significantly reduced pathological damage associated with *Plasmodium* infection and allowed the spleen to maintain a relative integrity of the germinal center. There was minimal immunoglobulin deposition on the glomerular capillary basement membranes in *pb*ANKA*-*infected mice, and there was glomerular capillary congestion in various groups, suggesting the occurrence of glomerulonephritis. In the brain, neurons and glial cells did not experience malaria pigment deposition. Notably, the structural integrity of myocardial fibers was conserved with regular alignment, and normal cardiomyocyte nuclei can be seen (Fig. 6D).

Overall, PEM-ARM was safe for in vivo application, which retained normal histological characteristics, and was shown to have a major role in protecting the vital organs in *pb*ANKA*-*challenged mice.

## Conclusion

Biomimetic erythrocyte membrane nanoliposomes (PEM-ARM) for the treatment of *pb*ANKA infection were successfully developed through a simple sonication-extrusion procedure with post-formation modification with the CLIPPKF peptide. PEM-ARM exhibited ideal physical and chemical properties, such as moderate particle size with a uniform PDI, high encapsulation efficiency, and good stability. PEM-ARM effectively captured free merozoites for intervening with repeated infections and selectively delivered ARM to iRBCs through interactions between CLIPPKF and the everted PS. This improved the concentration of cellular drugs, especially in trophozoite stages, to enhance the anti-malarial effects of ARM. By sequestering iRBCs, PEM-ARM significantly prevented cell apoptosis, hemozoin deposition, and inflammation activation in the lungs. Moreover, compared with the model group (PBS), PEM-ARM enhanced the ROS content in iRBCs and reduced the mitochondrial activity of *Plasmodium*. All these characteristics contributed to effectively attenuating *pb*ANKA infections.

In the fight against malaria, new antimalarial drugs with original mechanisms of action are urgently needed [47]. Given that the lipid metabolism of malaria parasites is crucial for intracellular growth as well as for propagation and transmission of the pathogen [48], the approach in this study was based on choline analogues to block *de novo* CDP-Cho-mediated phosphatidylcholine (PC) synthesis of the parasite in the blood stage at the cost of plasmatic choline [49]. Some researchers have suggested that nanocarriers with a size less than 80 nm may pass through membrane channels called new permeability pathways (NPPs) that emerge shortly after the invasion of *P. falciparum* in RBCs [50]. Thus, the advantage of a nano-delivery system in malaria treatment was based on the ability to enhance the interaction with RBCs [51]. The main goal of malaria treatment is to promote higher concentrations of drugs in intracellular parasitic vacuoles (PVs), where the parasite is hosted [52]. Here, we proposed a dual-targeted nanomimic of PEM-ARM that was capable of simultaneously targeting merozoites and infected erythrocytes in *pb*ANKA-infected mice for ultimate pathogen clearance. Furthermore, PEM-ARM theoretically provides a unique possibility of simultaneously encapsulating flavonoids, which have been confirmed to inhibit the *P. falciparum* fatty acid biosynthesis pathway [53]. As an extra benefit, the strategy of interfering with the parasite lifecycle (as is the case with nanomimics) and subsequently triggering an immune clearance represents a promising alternative to current drug treatment and vaccination strategies [54]. Unfortunately, while many receptor-ligand interactions have been characterized, their distinct functions and relative contributions to invasion are still not well stated. Moreover, other severe types of malaria, including cerebral malaria [55], and pregnancy-associated malaria (PAM) [56], and their response to the anti-malarial effects of PEM-ARM should be a topic of further research.

As such, the preparation of biomimetic nanoliposomes for multiple growth stages of *Plasmodium* is a promising strategy for anti-malarial therapy and has the potential to be applied to other parasitic infections.

## Supplementary Information

**Additional file 1.** Synthesis of DSPE-PEG_2000_-CLIPPKF. In vitro and in vivo experiment methods. **Fig. S1** Synthesis route of DSPE-PEG_2000_-CLIPPKF. **Fig. S2** Markers of erythrocyte membrane specificity. **Fig. S3** Construction of the HPLC conditions for ARM. **Fig. S4** Standard curve of ARM by the HPLC. **Fig. S5** Giemsa staining and calculation of infection rate. **Fig. S6** iRBCs-binding capacity. **Fig. S7** Neutralization of merozoites. **Fig. S8** TUNEL staining. **Fig. S9** Immunohistochemical analysis. **Fig. S10** iRBCs adhesion to frozen normal lung sections. **Fig. S11** Blood routine. **Fig. S12** Organ Coefficients (%). **Fig. S13** Hemolysis test in vitro. **Fig. S14** The nanoparticles tissue distribution. **Fig. S15** Serum biochemical parameters in mice at 24 h after administration. **Table S1** The EE and DL of the Lip-ARM, EM-ARM, and PEM-ARM.

## Data Availability

All data generated or analyzed during this study are included in this published article.
