# Correction: Erythrocyte membrane with CLIPPKF as biomimetic nanodecoy traps merozoites and attaches to infected red blood cells to prevent *Plasmodium* infection

**DOI:** 10.1186/s12951-026-05186-4

**Published:** 2026-11-07

**Authors:** Zhouqing He, Chuyi Yu, Ziyi Pan, Xiaobo Li, Xiangxiang Zhang, Qijing Huang, Xingcheng Liao, Jiaoting Hu, Feng Zeng, Li Ru, Wanlin Yu, Qin Xu, Jianping Song, Jianming Liang

**Affiliations:** 1 Artemisinin Research Center, The First Affiliated Hospital, Guangzhou University of Chinese Medicine, Guangzhou 510405, China; 2 Guangdong Provincial Hospital of Chinese Medicine, Guangzhou 510120, China; 3 Key Laboratory of Smart Drug Delivery, School of Pharmacy, Ministry of EducationFudan University, Shanghai 201203, China

**Correction: Journal of Nanobiotechnology (2023) 21:15**

**https://doi.org/10.1186/s12951-022-01709-x**

 In this article, incorrect fluorescence images for the f-ARM group and the Lip-ARM group in Fig. 6A were used. The incorrect figure, as well as the correct figure and caption have been included in this correction.

**Incorrect** **Fig. 6**

**Fig. 6:**
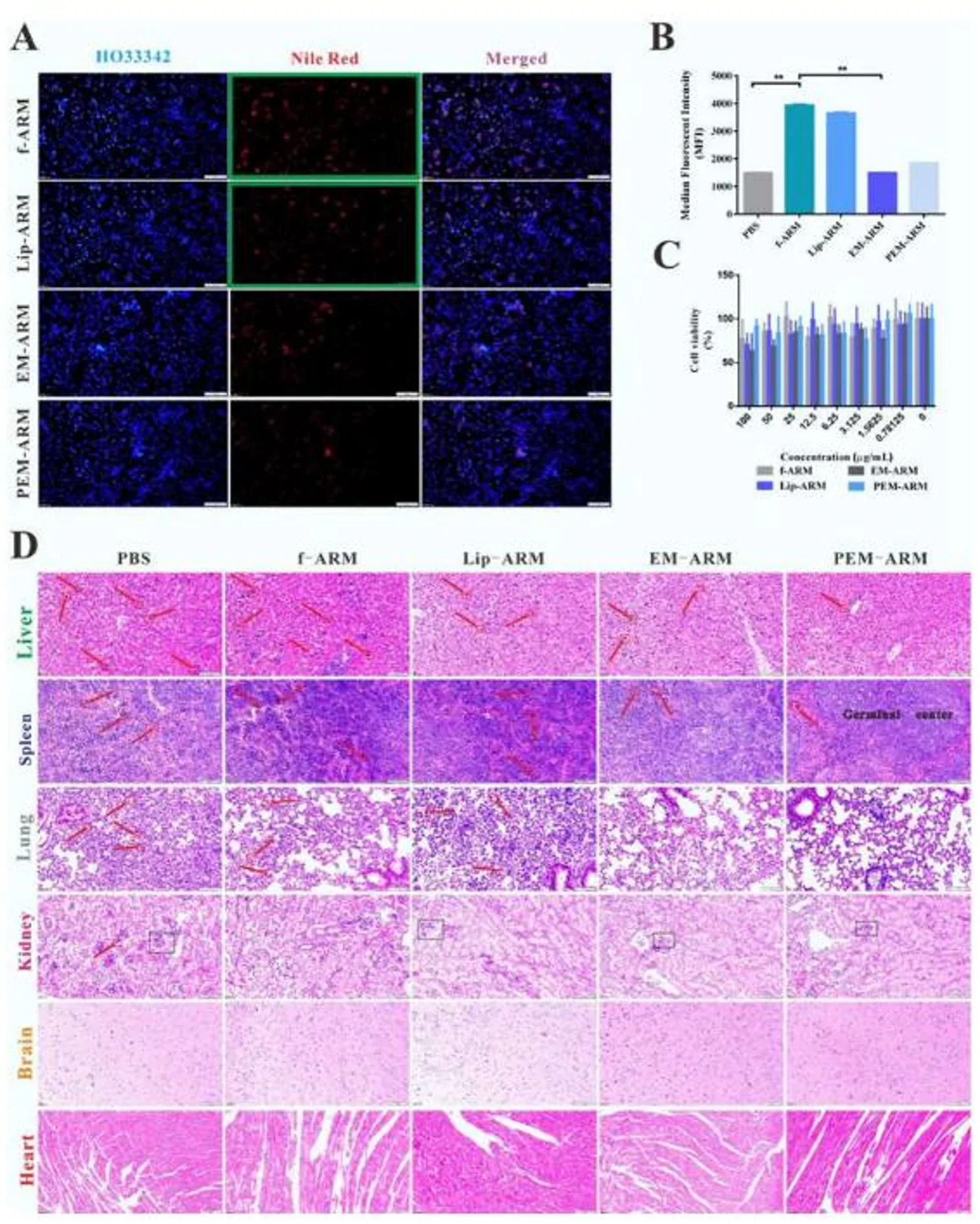
Characterizations of several measures of the safety of PEM-ARM. A Fluorescence images and B Median fluorescence intensity charts of MLE-12 cellular uptake after liposome treatment. Scale bar = 200 μm. Cell nuclei and the liposomes were labeled with HO (blue) and NR (red), respectively (*n* = 3, *P < 0.05 and ** *P* < 0.01). C MTT assay after MLE-12 cells were incubated with various concentrations of liposomes (*n* = 6, * *P* < 0.05 and ** *P* < 0.01). D H&E-stained slices of the liver, spleen, lung, kidney, brain, and heart. Scale bar = 200 μm, hemozoin (red arrow), glomerular capillary congestion sign (black frame)

**Correct** **Fig. 6**

**Fig. 6:**
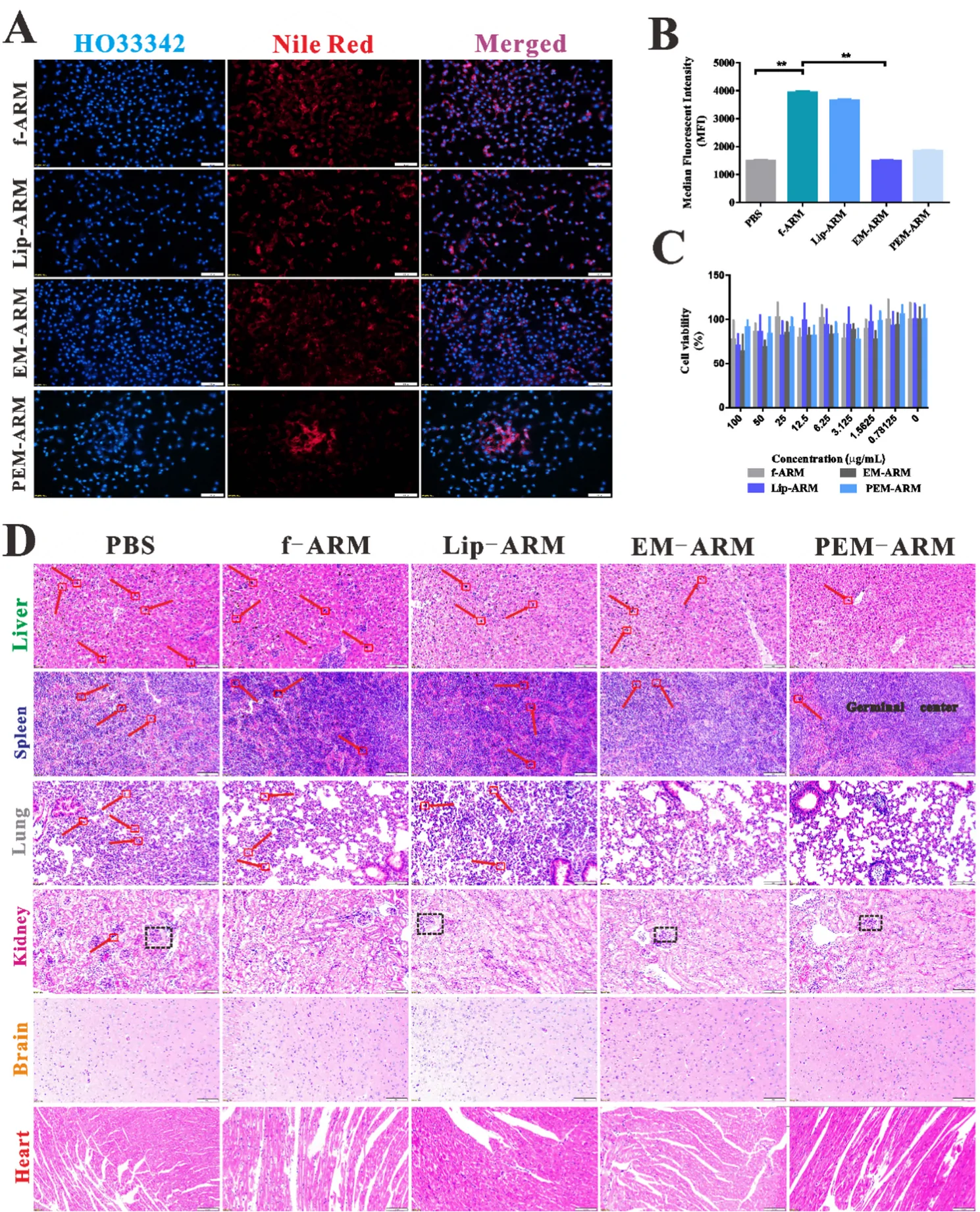
Characterizations of several measures of the safety of PEM-ARM. A Fluorescence images and B Median fluorescence intensity charts of MLE-12 cellular uptake after liposome treatment. Scale bar = 200 μm. Cell nuclei and the liposomes were labeled with HO (blue) and NR (red), respectively (*n* = 3, *P < 0.05 and ** *P* < 0.01). C MTT assay after MLE-12 cells were incubated with various concentrations of liposomes (*n* = 6, * *P* < 0.05 and ** *P* < 0.01). D H&E-stained slices of the liver, spleen, lung, kidney, brain, and heart. Scale bar = 200 μm, hemozoin (red arrow), glomerular capillary congestion sign (black frame)

The original article has been corrected.

