# Supplementary material for: Erythrocyte membrane with CLIPPKF as biomimetic nanodecoy traps merozoites and attaches to infected red blood cells to prevent Plasmodium infection
Source: J Nanobiotechnology. 2023 Jan 16;21:15. doi: 10.1186/s12951-022-01709-x (PMC9841648; doi:10.1186/s12951-022-01709-x)
Supplement: Supplementary file 1 — Additional file 1. Synthesis of DSPE-PEG2000-CLIPPKF. In vitro and in vivo experiment methods. Fig. S1 Synthesis route of DSPE-PEG2000-CLIPPKF. Fig. S2 Markers of erythrocyte membrane specificity. Fig. S3 Construction of the HPLC conditions for ARM. Fig. S4 Standard curve of ARM by the HPLC. Fig. S5 Giemsa staining and calculation of infection rate. Fig. S6 iRBCs-binding capacity. Fig. S7 Neutralization of merozoites. Fig. S8 TUNEL staining. Fig. S9 Immunohistochemical analysis. Fig. S10 iRBCs adhesion to frozen normal lung sections. Fig. S11 Blood routine. Fig. S12 Organ Coefficients (%). Fig. S13 Hemolysis test in vitro. Fig. S14 The nanoparticles tissue distribution. Fig. S15 Serum biochemical parameters in mice at 24 h after administration. Table S1 The EE and DL of the Lip-ARM, EM-ARM, and PEM-ARM. [file 12951_2022_1709_MOESM1_ESM.docx]

**Additional file 1**

**Erythrocyte membrane with CLIPPKF as biomimetic nanodecoy traps merozoites and attaches to infected Red Blood Cells to prevent *Plasmodium* infection**

Zhouqing He1,†, Chuyi Yu1, Ziyi Pan1, Xiangxiang Zhang1, Qijing Huang1, Xingcheng Liao1, Jiaoting Hu1, Feng Zeng^1^, Li Ru^1^, Wanlin Yu^2^, Qin Xu^1^, Jianping Song^1^, Jianming Liang^1,3,*^

1Artemisinin Research Center, The First Affiliated Hospital, Guangzhou University of Chinese Medicine, Guangzhou 510405, China.

2Guangdong Provincial Hospital of Chinese Medicine, Guangzhou 510120, China.

^3^Key Laboratory of Smart Drug Delivery, Ministry of Education, School of Pharmacy, Fudan University, Shanghai 201203, China.

*Corresponding author

E-mail address: [liangjianming@gzucm.edu.cn](mailto:040163@gzucm.edu.cn) (Jianming Liang)

**Supporting experimental methods**

**Synthesis of DSPE-PEG_2000_-CLIPPKF**

5 mg DSPE-PEG_2000_-Mal was dissolved in 900 μL tetrahydrofuran and placed in a 25 mL eggplant-shaped bottle with a constant speed of 250 rpm rotation. To ensure completely reaction of DSPE-PEG_2000_-Mal, the CLIPPKF peptide plunged into this reaction was weighed in terms of the molar ratio of DSPE-PEG_2000_-Mal to CLIPPKF of 2 : 1, and dissolved in 100 μL of PBS (pH = 8.0) containing 0.1 % EDTA and then slowly dripped into the bottle. We added triethylamine to catalyze the reaction with a triethylamine-CLIPPKF molar ratio of 3 : 1, and kept the whole system away from light for 24 h at room temperature. After reaction, the tetrahydrofuran was removed by rotary evaporation, and 1 mL of ddH_2_O was recommended to hydrate the final product. The gathered solution was placed in a dialysis bag with 2000 Da molecular weight, and dialyzed for 24 h to take away the extra CLIPPKF. The formed product DSPE-PEG_2000_-CLIPPKF was characterized and analyzed by the high-performance liquid chromatography (HPLC) and lyophilized for storage.

**Characterized protein CD47 analysis by Western Blotting**

Proteins in samples were separated by SDS-PAGE as previously described，we transferred the separated proteins to the PVDF blotting membrane at 200 mA for 2 h, and blocked this membrane with 5% skimmed milk powder solution for 90 min. The PVDF blotting membrane was placed in the Western primary antibody dilution buffer containing primary antibody CD47, incubated overnight at 4 °C and shaken. Subsequently, the membrane was rinsed three times with PBST solution, put into a Western secondary antibody dilution buffer containing HRP-conjugated secondary antibody, and incubated at room temperature for 2 h with shaking. We washed the PVDF blotting membrane three times with PBST solution, and finally finished the detection of protein CD47 using a WB substrate kit.

**Determination of glycoproteins on the erythrocyte membrane**

Fluorescently labeled wheat germ agglutinin (WGA) was recommended to characterize the glycoproteins on the erythrocyte membrane surface. Briefly, the Lip-ARM, RBCm, EM-ARM or PEM-ARM was incubated with 1 μg mL^-1^ Texas Red^®^-X-conjugated WGA at room temperature for 10 min away from light. When the co-incubation finished, the solution was put into a dialysis bag with 100 KD molecular weight, and dialyzed overnight at 4 °C to remove the free WGA. The Lip-ARM and RBCm solutions were set as the negative and positive control group, respectively. The fluorescence intensity (excitation max: 595 nm, emission max: 615 nm) was detected by a microplate reader.

**Determination of encapsulation efficiency and drug loading capacity**

The encapsulation efficiency (EE) and drug loading capacity (DL) of ARM were determined by the HPLC, and its conditions were confirmed as follows: the mobile phase was a mixture of methanol, water and acetic acid with a ratio of 85.4 : 13.6 : 1 at a flow rate of 1.0 mL/min using a C18 column (150 × 4.6 mm, 5 μm, Agilent, USA) isocratic elution in the temperature of 25 ^o^C, the detection wavelength was 254 nm with an injection volume of 50 μL. We established the standard curve equation of ARM by taking concentration as the abscissa and peak area as the ordinate, and the EE and DL of ARM were calculated as Equation (1) and (2), respectively:

 (1)

 (2)

Where *p_ARM_* (mg mL^-1^) represented mass concentration of ARM, *V* (mL) represented the solution volume, *m_total_* (mg) was the weight of ARM input and *m_NP_* (mg) was the weight of total nanoliposomes.

**Giemsa staining and calculation of infection rate**

The tail tip of mice should be pricked with a sharp sterile needle, two drops of blood were placed at one end of the clean and grease-free glass slide. The thin blood film was made immediately by placing the smooth leading edge of a second slide in the central drop of blood, adjusting the angle and holding the edges of the slide in the same time, smearing the blood with a swift and steady sweep along the surface.

The thin film should be dried thoroughly otherwise it may be washed away during staining. The thin film was then stained by Giemsa dye solution according to the instruction manual, the number of parasitized red blood cells per 1000 red blood cells should be counted under oil immersion at a magnification of ×1000, and infection rate was iRBCs number divided by the total number of red blood cells.

**iRBCs adhesion to frozen normal lung sections**

iRBCs Adhesion assays in vitro to lung tissue were conducted in accordance with interrelated literature with slightly modified [1], Percoll-purified iRBCs were placed in RPMI 1640 containing 1% SYBR Green I with quantities of drugs (PBS, f-ARM, Lip-ARM, EM-ARM, or PEM-ARM) for 30 min incubation, we resuspended them at RPMI 1640 medium without any dyes after centrifugal and washing procedures. In the meantime, 5 μm cryosections of normal lung tissue were immersed in 1640 medium containing HO. Subsequently, the SYBR Green I-labeled iRBCs were incubated on unfixed sets of three HO-labeled cryosections of normal lung tissue at room temperature. The Adhesion was carried out for 1 h, with successive shake of 40 rpm, followed by gentle washing in PBS to remove unbound iRBCs. We covered the cryosections with autofluorescence quencher for 1 h incubation at 37 ^o^C, washed gently three times in 1 × PBS, and observed the adhesion under a fluorescence microscope after glycerin gelatin-mounted.

**Hemolysis test in vitro**

The purified normal erythrocytes were suspended in PBS with a concentration of 6% (v/v), and the f-ARM, Lip-ARM, EM-ARM, or PEM-ARM was adjusted to a concentration of 0.5 mg mL^-1^ with 1 × PBS. The water, PBS and drugs were set as shown in Tab. 1.

| **Tab. 1 Hemolysis test with various samples treatment** | | | | | |
| --- | --- | --- | --- | --- | --- |
| Group | Number | 6% RBC solution  (mL) | 1 × PBS  (mL) | H_2_O  (mL) | Sample  (mL) |
| f-ARM | 1 | 1 | 0.8 | 0 | 0.2 |
| Lip-ARM | 2 | 1 | 0.8 | 0 | 0.2 |
| EM-ARM | 3 | 1 | 0.8 | 0 | 0.2 |
| PEM-ARM | 4 | 1 | 0.8 | 0 | 0.2 |
| H_2_O | 5 | 1 | 0 | 1 | 0 |
| 1 × PBS | 6 | 1 | 1 | 0 | 0 |

The mixed solutions were blended uniformly and incubated at 37 ^o^C with a speed of 100 rpm in a constant temperature shaker. We took the supernatant away at the time point of 0, 0.5, 1, 2, 3, 4, and 5 h, and added them to 96 well plate with 50 μL per well. Three multiple wells were set in each group, and the OD values of each well at 545 nm were measured. The water or PBS group was set as the positive or negative control group, respectively. The hemolytic rate was calculated according to the Equation (3)

 (3)

Where *OD_sample_* represented the absorbance of the sample at 545 nm, *OD_positive_* represented the absorbance of the water at 545 nm, *OD_negative_* was the absorbance of the PBS at 545 nm.

**Biocompatibility** **research**

The *pb*ANKA-challenged mice received an intravenous injection of NR-labeled liposomes at day 5 with an average infection rate was 5%. We collected the isolated organs (heart, liver, spleen, lung, and kidney) when mice were sacrificed at 24 h, and the near-infrared fluorescence in vivo imaging system was employed to investigate the nanoparticles tissue distribution as an index of potential toxic side effects. To explore whether there exists any toxic effect after administration, the normal C57BL/6 mice received an intravenous injection of f-ARM, Lip-ARM, EM-ARM, or PEM-ARM containing an equivalent ARM content of 2.5 mg·kg^-1^, and PBS as a control. After 24 h, whole blood was collected through the orbital vein, and serum was separated by centrifugation at 3200 rpm at room temperature for 10 min. Subsequently, the general histology and serum enzymatic parameters were measured to evaluate the toxic effects at 24 h after administration.

**References**

1. Yosaatmadja F, Andrews KT, Duffy MF, Brown GV, Beeson JG, Rogerson SJ: Characterization of VAR2CSA-deficient Plasmodium falciparum-infected erythrocytes selected for adhesion to the BeWo placental cell line. Malaria J. 2008;7(1):51.

**Additional file 1 Figures**


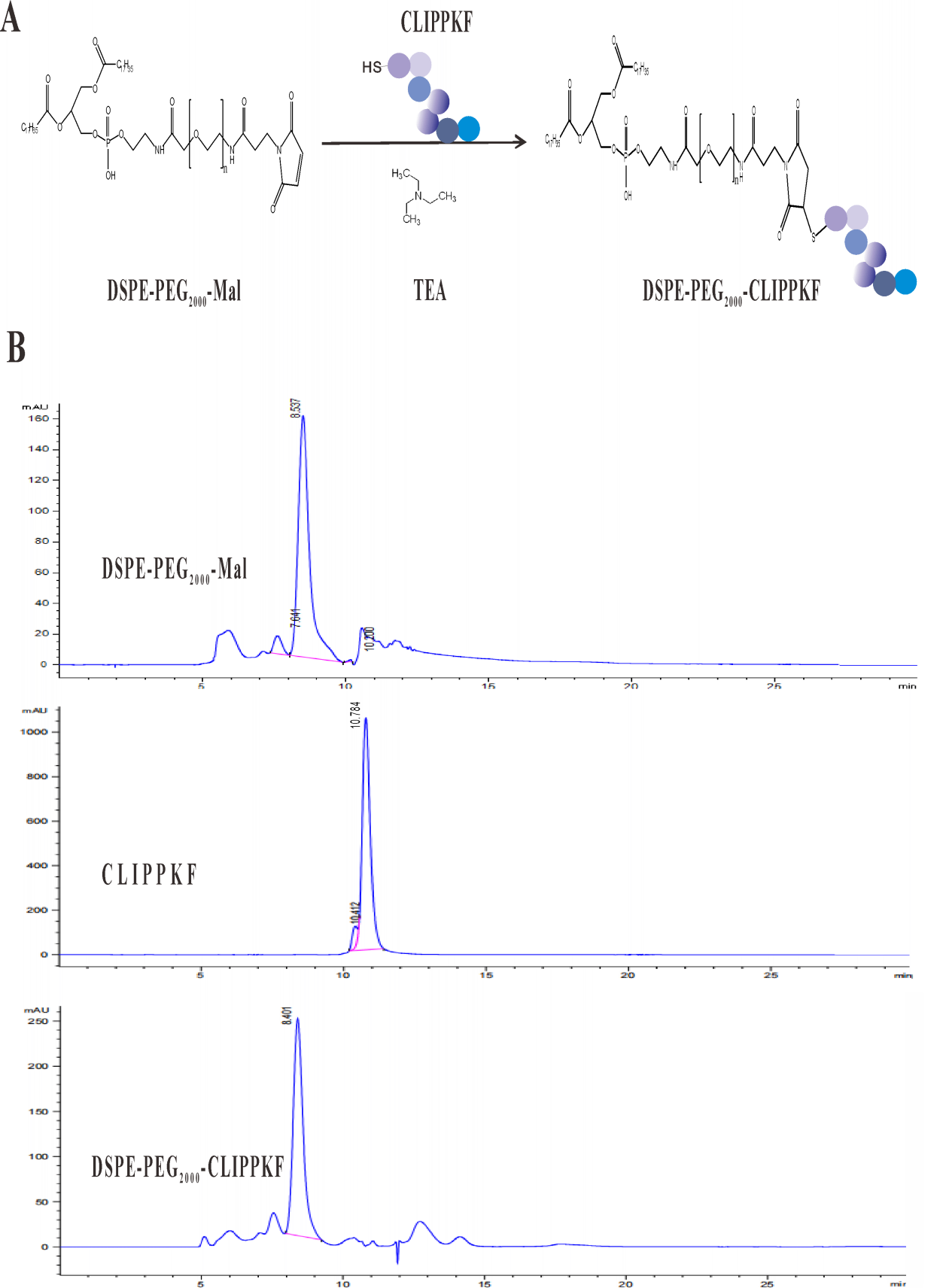


**Fig. S1** Synthesis of DSPE-PEG_2000_-CLIPPKF. (A) Scheme of the synthesis process. (B)The HPLC diagram of DSPE-PEG_2000_-Mal, CLIPPKF, and DSPE-PEG_2000_-CLIPPKF.


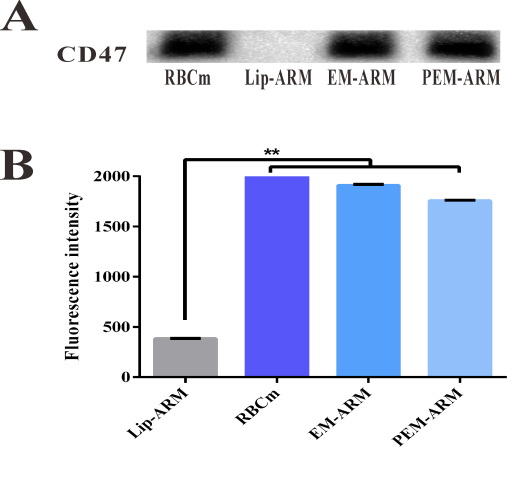


**Fig. S2** Markers of erythrocyte membrane specificity. (A) Western blotting of characterized protein CD47 in RBCm, Lip-ARM, EM-ARM and PEM-ARM. (B) The fluorescence intensity of glycosylated membrane proteins in RBCm, Lip-ARM, EM-ARM and PEM-ARM was determined with Texas Red^®^-X-conjugated WGA (n = 3, ^*^ *P* < 0.05 and ^**^*P* < 0.01).


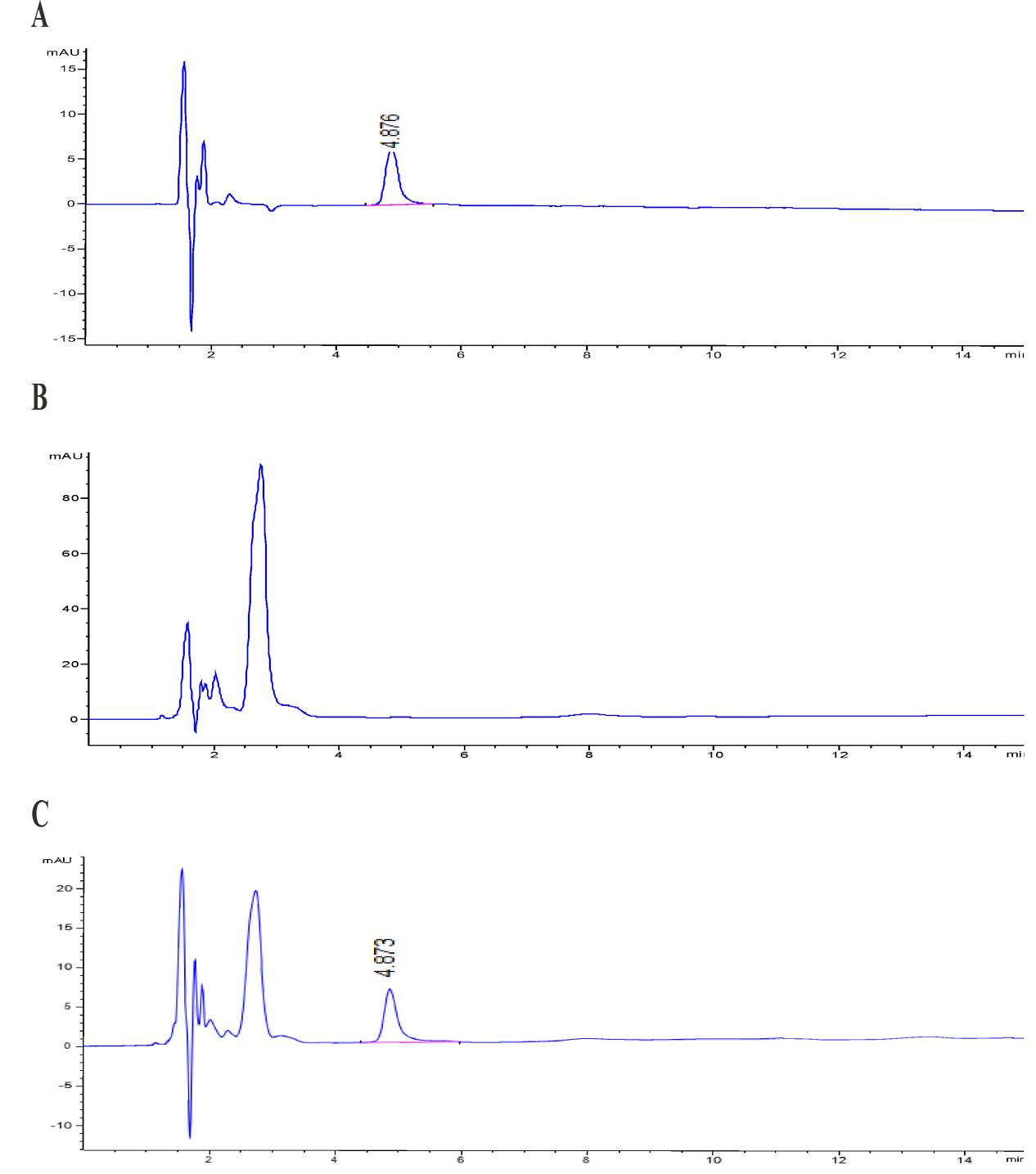


**Fig. S3** Construction of the HPLC conditions for ARM. (A) ARM had an absorption peak at 4.876 min at 254 nm. (B) There was no absorption peak in CHO and EPC under the same conditions. (C) The absorption peak reappeared at 4.873 min with a blend of ARM, CHO, and EPC.


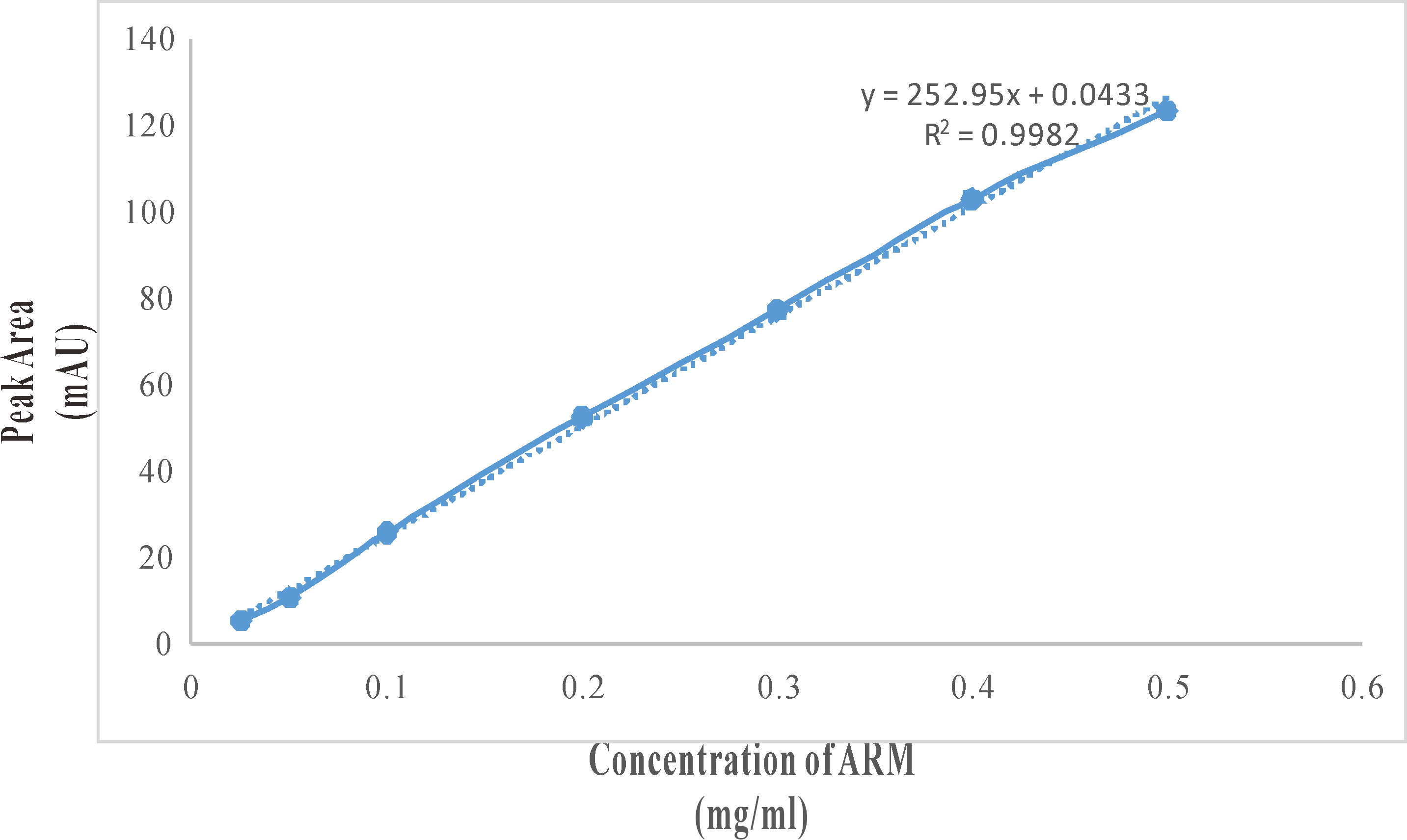


**Fig. S4** Standard curve of ARM by the HPLC**.** The abscissa represented concentration of ARM, and the ordinate represented the peak area.

**Tab. S1** The EE and DL of the Lip-ARM, EM-ARM, and PEM-ARM

|  | EE (%) | DL (%) |
| --- | --- | --- |
| Lip-ARM | 93.41±0.04 | 4.36±0.09 |
| EM-ARM | 93.40±0.06 | 4.18±0.18 |
| PEM-ARM | 93.42±0.03 | 4.09±0.26 |


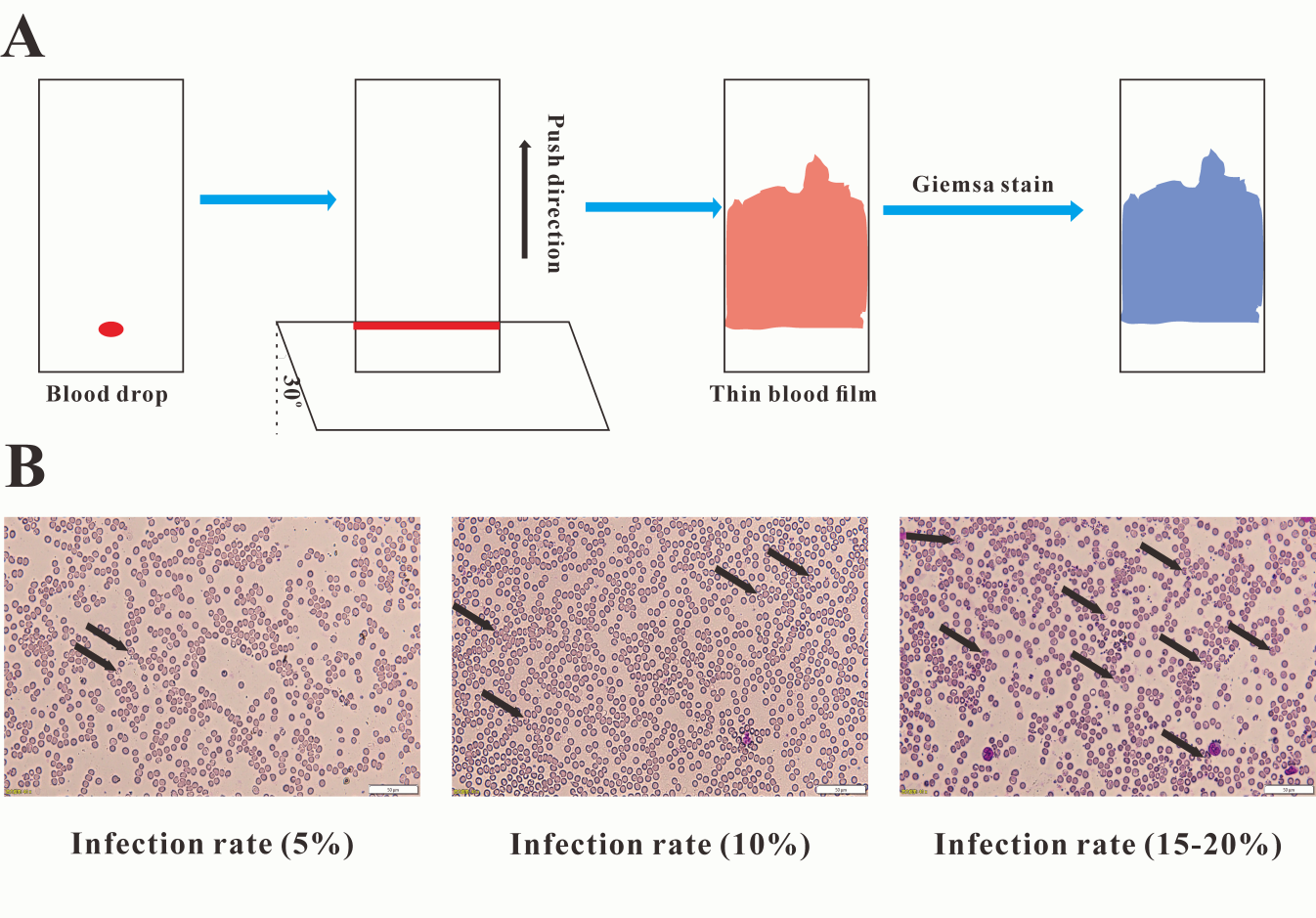


**Fig. S5** Giemsa staining and calculation of infection rate. (A) Schematic diagram of thin blood film making and Giemsa staining process. (B) Microscopic images of infected red blood cells with different infection rates, iRBCs (black arrow). Scale bar = 50 μm.

**Fig. S6** iRBCs-binding capacity. Normal erythrocytes, rings, trophozoites, and schizonts took up liposomes in mice with a 5% infection rate.(n = 3, ^*^ *P* < 0.05 and ^**^ *P* < 0.01).


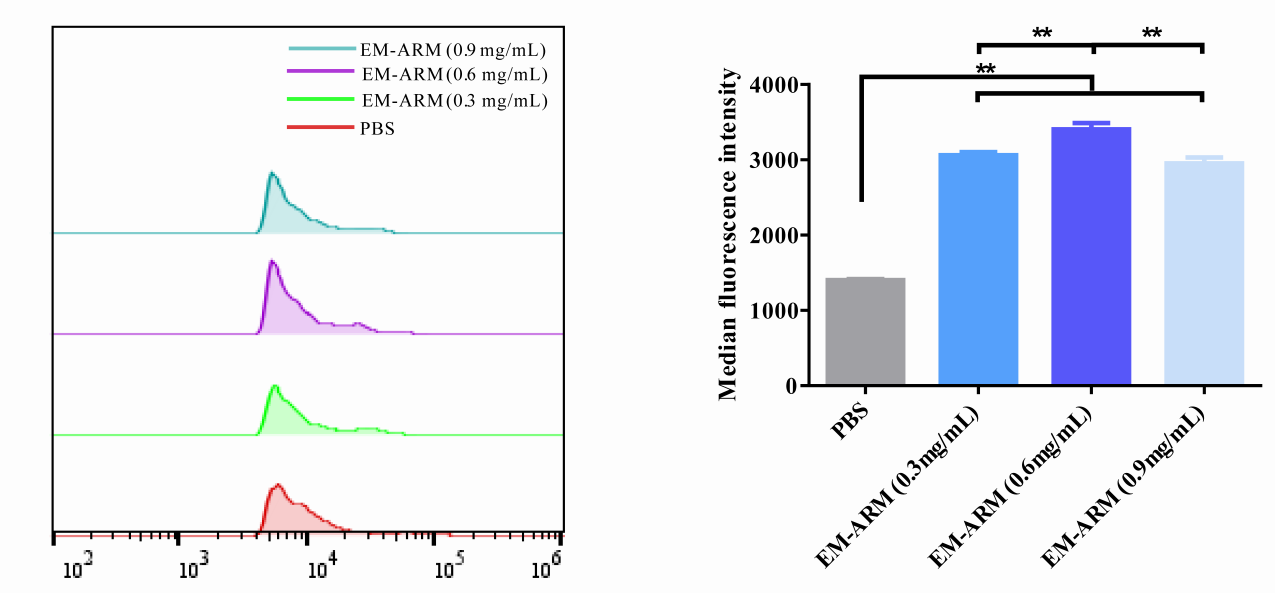


**Fig. S7** Neutralization of merozoites. Uptake of EM-ARM with different membrane protein concentrations by merozoites in *pb*ANKA-infected mice (n = 3, ^*^ *P* < 0.05 and ^**^*P* < 0.01).


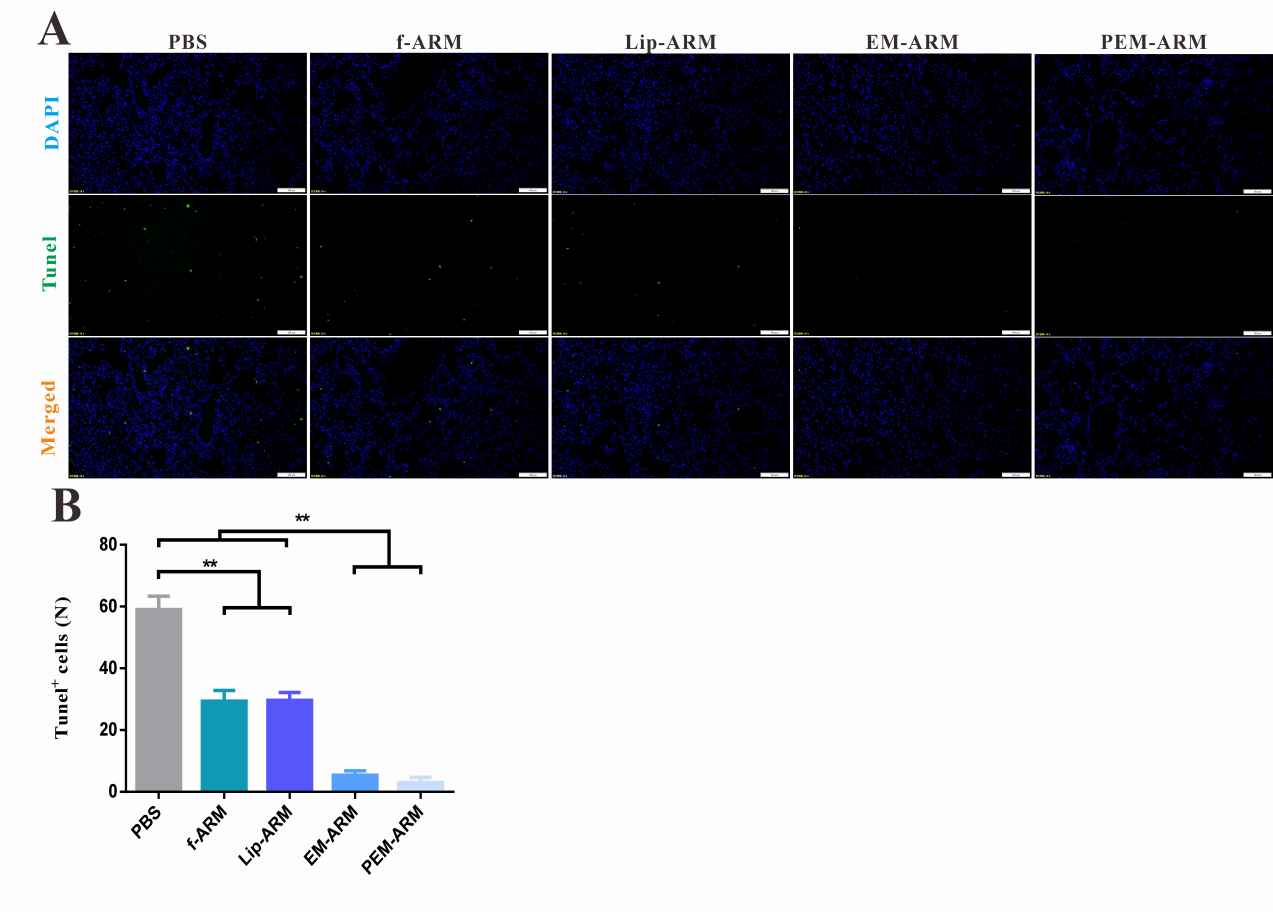


**Fig. S8** TUNEL staining. (A) Fluorescent images and (B) column chart of TUNEL staining of lung tissues, nuclei (blue) and TUNEL positive (green) pulmonary epithelial cells. Scale bar = 100 μm (n = 3, ^*^ *P* < 0.05 and ^**^ *P* < 0.01).


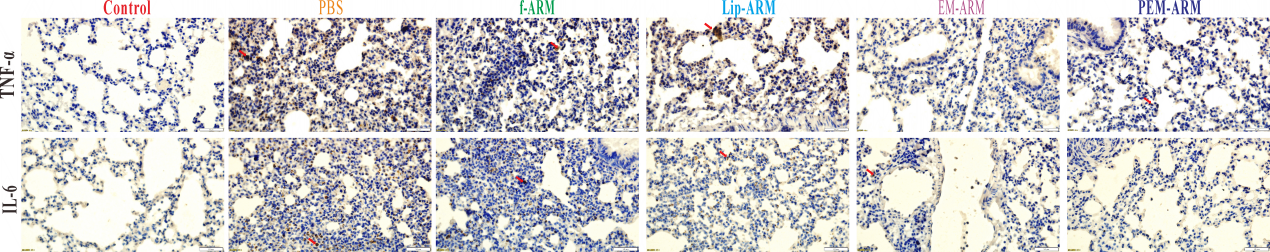


**Fig. S9** Immunohistochemical analysis. Levels of TNF-ɑ and IL-6 in lung tissues were examined, hemozoin (red arrow). Scale bar = 50 μm.


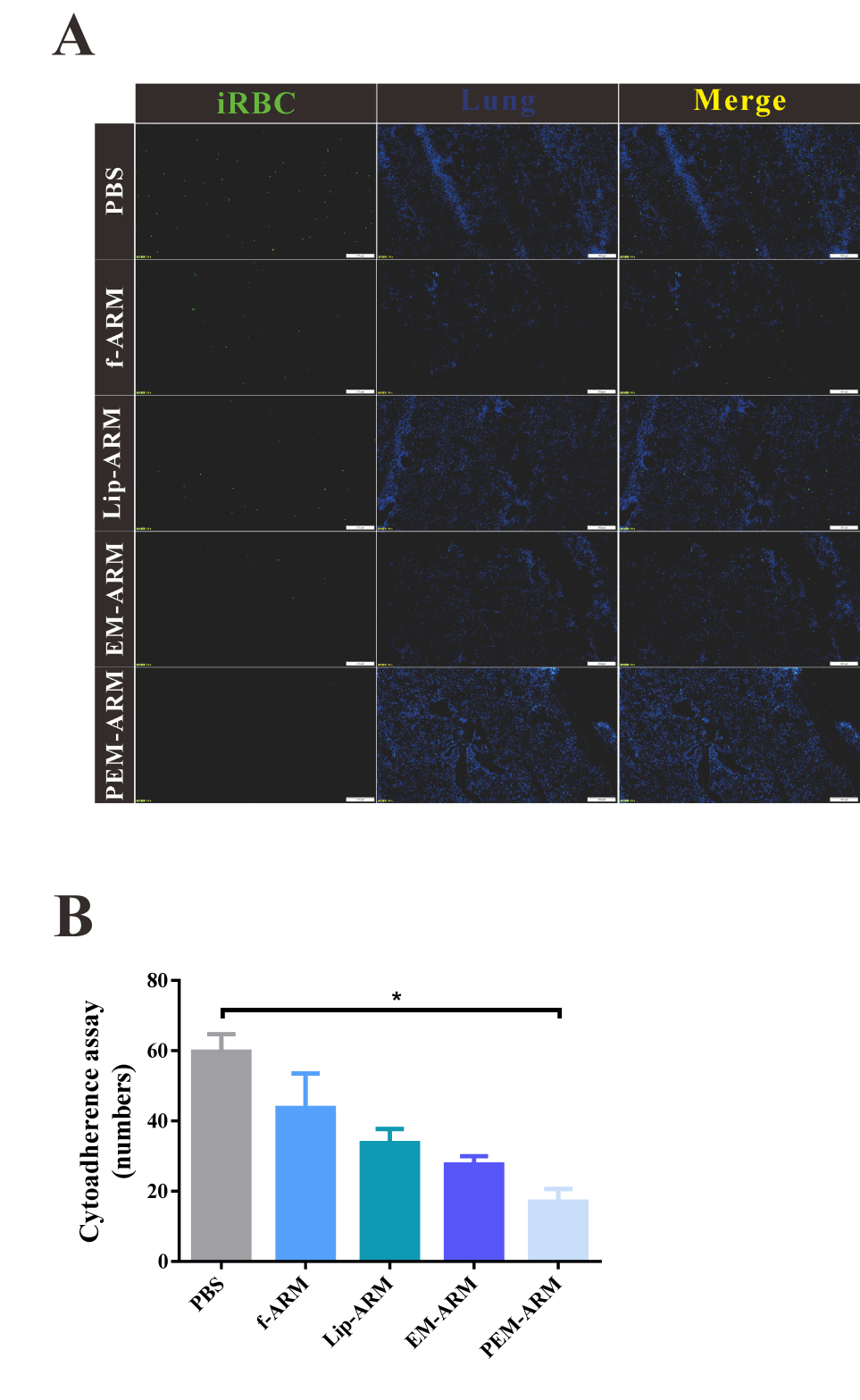


**Fig. S10** iRBCs adhesion to frozen normal lung sections. (A) Fluorescent images and (B) column chart of iRBCs adhesion assay to normal lung tissues, pulmonary epithelial cells (blue) and iRBCs (green). Scale bar = 200 μm (n = 3, ^*^*P* < 0.05 compared with the PBS group).

**Fig. S11** Blood routine. Comparison in the numbers of RBCs, hemoglobin (HGB), hematocrit (HCT), and platelets (PLT) (n = 4, ^*^ *P* < 0.05 and ^**^ *P* < 0.01).

**Fig. S12** Organ Coefficients (%). (A) Liver, (B) spleen, (C) lung, (D) heart, (E) kidney and (F) brain (n = 4, ^*^ *P* < 0.05 and ^**^ *P* < 0.01).

**Fig. S13** Hemolysis test in vitro. Which includes OD_545_ values at different time points after incubation with various drugs.


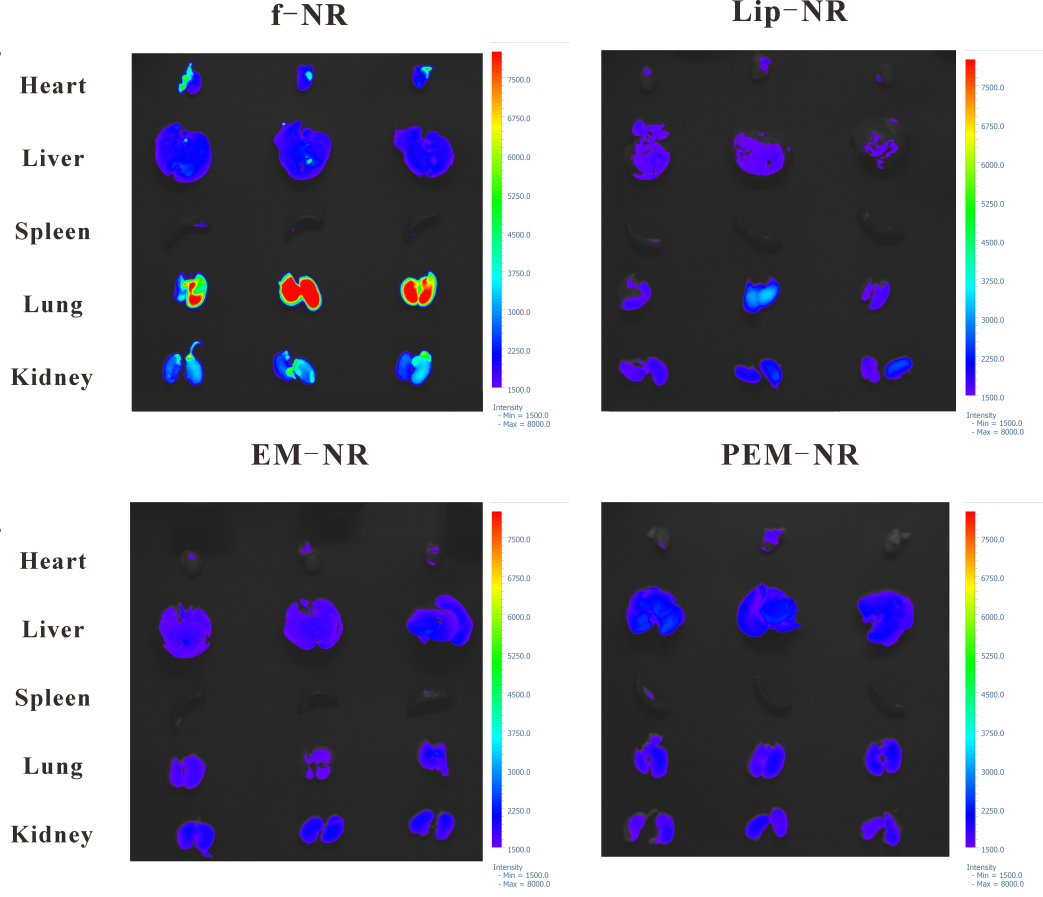


**Fig. S14** The nanoparticles tissue distribution. Which includes the in vivo imaging of the accumulation of NR at various organs.


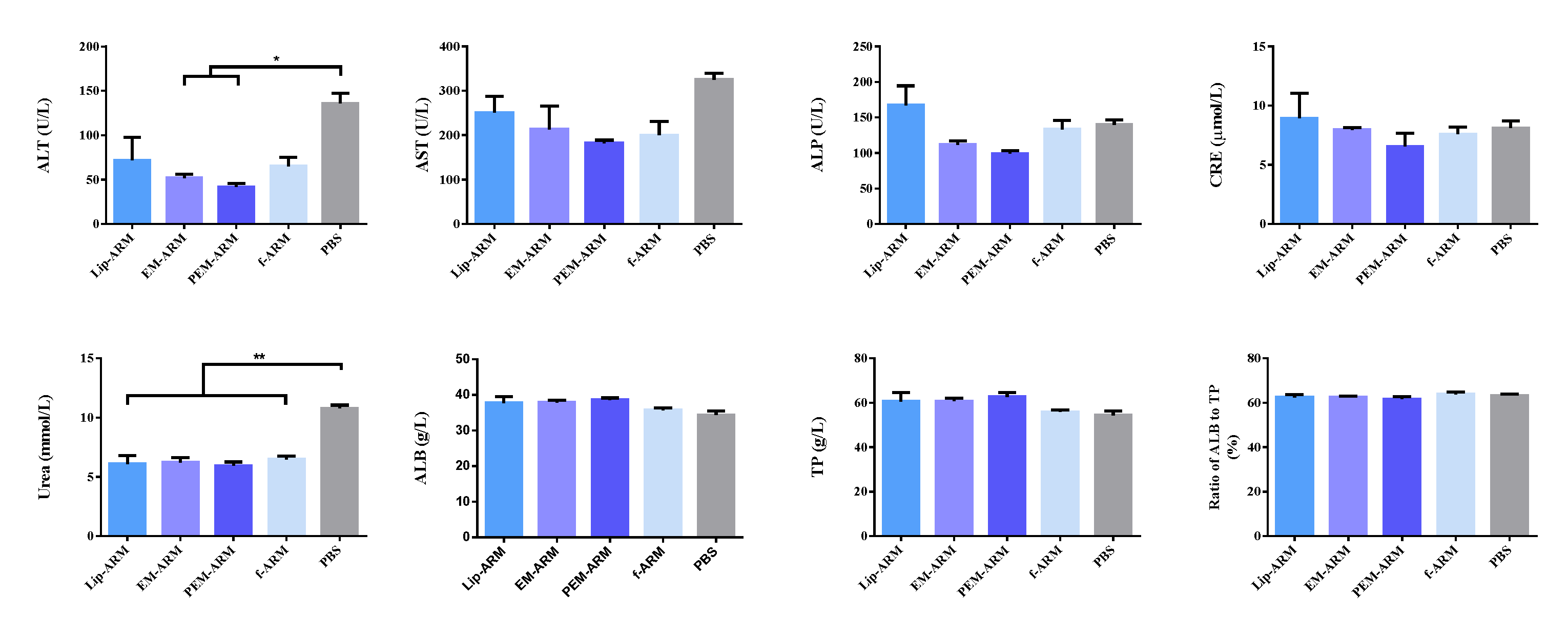


**Fig. S15** Serum biochemical parameters in mice at 24 hours after administration. Comparison in the contens of alanine aminotransferases (ALT), aspartate aminotransferase (AST), alkaline phosphatase (ALP), creatinine (CRE), urea，albumin (ALB), total protein (TP) and the ratio of ALB to TP (n = 3, ^*^ *P* < 0.05 and ^**^ *P* < 0.01).
